# Integrated Analysis of miR-430 on Steroidogenesis-Related Gene Expression of Larval Rice Field Eel *Monopterus albus*

**DOI:** 10.3390/ijms22136994

**Published:** 2021-06-29

**Authors:** Lihan Zhang, Qiushi Yang, Weitong Xu, Zhaojun Wu, Dapeng Li

**Affiliations:** 1 College of Fisheries, Huazhong Agricultural University, Wuhan 430070, China; 2014zhang@webmail.hzau.edu.cn (L.Z.); y357446@webmail.hzau.edu.cn (Q.Y.); xuweitong@webmail.hzau.edu.cn (W.X.); wuzj@webmail.hzau.edu.cn (Z.W.); 2 Engineering Research Center of Green Development for Conventional Aquatic Biological Industry in the Yangtze River Economic Belt, Ministry of Education, Wuhan 430070, China; 3 Hubei Provincial Engineering Laboratory for Pond Aquaculture, Wuhan 430070, China; 4 Freshwater Aquaculture Collaborative Innovation Center of Hubei Province, Wuhan 430070, China

**Keywords:** miR-430, steroidogenesis, *Monopterus albus*, early stage

## Abstract

The present study aims to reveal the mechanism by which miR-430s regulate steroidogenesis in larval rice field eel *Monopterus albus*. To this end, *M. albus* embryos were respectively microinjected with miRNA-overexpressing mimics (agomir430a, agomir430b, and agomir430c) or miRNA-knockdown inhibitors (antagomir430a, antagomir430b, and antagomir430c). Transcriptome profiling of the larvae indicated that a total of more than 149 differentially expressed genes (DEGs) were identified among the eight treatments. Specifically, DEGs related to steroidogenesis, the GnRH signaling pathway, the erbB signaling pathway, the Wnt signaling pathway, and other pathways were characterized in the transcriptome. We found that steroidogenesis-related genes (hydroxysteroid 17-beta dehydrogenase 3 (*17β-hsdb3*), hydroxysteroid 17-beta dehydrogenase 7 (*17β-hsdb7*), hydroxysteroid 17-beta dehydrogenase 12 (*17β-hsdb12*), and cytochrome P450 family 19 subfamily a (*cyp19a1b*)) were significantly downregulated in miR-430 knockdown groups. The differential expressions of miR-430 in three gonads indicated different roles of three miR-430 (a, b, and c) isoforms in regulating steroidogenesis and sex differentiation. Mutation of the miR-430 sites reversed the downregulation of cytochrome P450 family 17 (*cyp17*), *cyp19a1b*, and forkhead box L2 (*foxl2*) reporter activities by miR-430, indicating that miR-430 directly interacted with *cyp17*, *cyp19a1b*, and *foxl2* genes to inhibit their expressions. Combining these findings, we concluded that miR-430 regulated the steroidogenesis and the biosynthesis of steroid hormones by targeting *cyp19a1b* in larval *M. albus*. Our results provide a novel insight into steroidogenesis at the early stage of fish at the molecular level.

## 1. Introduction

Steroidogenesis plays pivotal roles in regulating physiological divergence, gonadal differentiation, and sex determination [1]. In the past decades, significant advances have been achieved in the genetics, molecular biology, and biochemistry of genes related to steroidogenesis and sexual differentiation in mammals and teleosts [2,3,4]. Steroidogenic enzymes consisting of the cytochrome P450 heme-containing proteins and the hydroxysteroid dehydrogenases [5,6] have been reported to alter the proportions as well as absolute and relative concentrations of sex hormones, thus resulting in sex differentiation [7,8]. Moreover, several transcription factors (SRY-box transcription factor 9, *sox9*; forkhead box L2, *foxl2*; steroidogenic factor 1, *sf-1*; Wilms tumor 1, *wt1*) have been found to be involved in the sex determination process [9,10,11]. Cytochrome P450 family 19 subfamily a (*cyp19a*) has been identified to transform androgens into estrogens, which can be regulated by *foxl2* together with other partners *sf-1* [12,13]. Cytochrome P450 family 17 (*cyp17*) as the steroid synthase transcriptional activator has been reported to be negatively regulated by the interaction of *foxl2* and *sf-1* in granulosa cells [14]. Although some key genes have been identified, the critical network in sex steroid hormone synthesis as well as reproductive processes is still unclear and needs to be further investigated.

MicroRNAs (miRNAs), a class of 20–25 nt noncoding RNAs, induce degradation or translation inhibition of target mRNAs by binding to the 3′UTR of target genes [15]. The miRNAs have been reported to be involved in the processes of gametogenesis, embryogenesis, steroidogenesis, fertilization, sex differentiation, and reproduction in mammals [16,17]. For instance, cytochrome P450 11 beta (*cyp11b*) and *cyp19a* are regulated by miR-10b and miR-378, respectively [16,18]. The miRNA regulates ovarian hormone synthesis by targeting steroidogenic mRNAs [19,20,21]. Similarly, several functional miRNA–target gene pairs such as miR-456/anti-Müllerian hormone (*amh*), miR-138/*cyp17a2*, and miR-20a/doublesex-mab3-related transcription factor1 (*dmrt1*) have been identified, revealing the critical roles of miRNA in steroidogenesis in fish [22]. The microRNA-430 family (miR-430s), as one of the most highly expressed miRNAs during fish development, regulates a number of target genes in embryogenesis [23,24]. The high expression of miR-430s during the early development of fish plays a vital role in the removal of maternal transcripts [25,26]. Moreover, the injection of miR-430 has been reported to rescue the brain defects in MZ*dicer* mutants of zebrafish, revealing crucial roles for miR-430 during morphogenesis in fish [25]. In fish, three isoforms of miR-430 (a, b, c) have been identified to be homologous in the 3′ region and the target recognition sites at the 5′ end of miR-430 (a, b, c), for their target genes (such as *C1q-like* genes) are identical. [24,27,28]. miR-430 has been reported to repress primordial germ cells (PGCs) development by directly targeting *C1q-like* in early embryos of *Carassius auratus*, indicating an essential role of miR-430 in embryogenesis [24]. Apart from its involvement in embryogenesis and morphogenesis, miR-430 is vital for the reproductive development, but its regulatory role in steroidogenesis remains unknown.

Rice field eel *Monopterus albus*, a nutritious freshwater fish, is a hermaphroditic protogynous species that undergoes natural sex reversal [29]. Our recent studies reveal some potential mechanism of steroidogenesis, ovary development, and sex differentiation by gene cloning and RNA-seq analysis in rice field eel [9,10,11,30,31]. Particularly, the differential expression of miR-430 was observed in three genders of *M. albus*, revealing that miR-430 might be involved in the steroidogenesis of this species [30]. Furthermore, foxl2 has been reported to be one of the target genes of miR-430b in rice field eel [11]. Given the critical roles of *cyp19* and *foxl2* in steroidogenesis, we speculated that miR-430 might indirectly mediate *cyp19* by targeting *foxl2* to regulate steroidogenesis in rice field eel. Therefore, the present study aims to investigate the effects of miR-430 on steroidogenesis at transcriptional and molecular levels in fish. To this end, *Monopterus albus* was microinjected with three miR-430s, and the samples were subjected to RNA-seq analysis and qPCR validation. Luciferase reporter gene was used to further validate the relationship between miR-430 and steroidogenesis-related genes, and miR-430 precursors were predicted and cloned. Our findings provide a better understanding of the roles of miR-430 in rice field eel steroidogenesis and contribute to identifying novel targets for sex reversal.

## 2. Results

### 2.1. Embryonic and Postembryonic Development of Monopterus albus

The periods of embryonic development of rice field eel were exhibited in Figure S1. The fertilized eggs of *M. albus* was sphere-like with yellow color after absorbing water. At water temperatures of 28–31 °C, embryonic development was divided into six stages: zygote period, cleavage period, blastula period, gastrula period, hatching prophase, and hatching (Figure S1). In particular, brain primordium and somite of *M. albus* occurred at the 50% epiboly stage (Figure S1f; 1.3 dpf). Sampling occurred on day 7 (Figure S1l; 2 d after hatching). Taken together, our data indicated that the brain of rice field eel occurred on the sampling day, which supported structural observation for subsequent analysis of the transcriptome.

### 2.2. Transcriptome Sequencing Analysis

#### 2.2.1. miR430 Expression Level after miR-430 Microinjection of *M. albus*

We determined the corresponding expression levels of miR-430s after various microinjections of miR-430 in rice field eel. As shown in Figure S2, the expression level of mir-430 was significantly increased after agomir injection with the three isoforms of mir-430 in *M. albus* compared to the negative control (agomirNC). In contrast, the expression level of miR-430 was significantly decreased after the antagomir injection, compared to the negative control (antagomirNC). Taken together, these results indicated that miR-430 expression was significantly altered after miR-430 microinjection.

#### 2.2.2. Identification and Enrichment Analysis of DEGs

**Figure 1:**
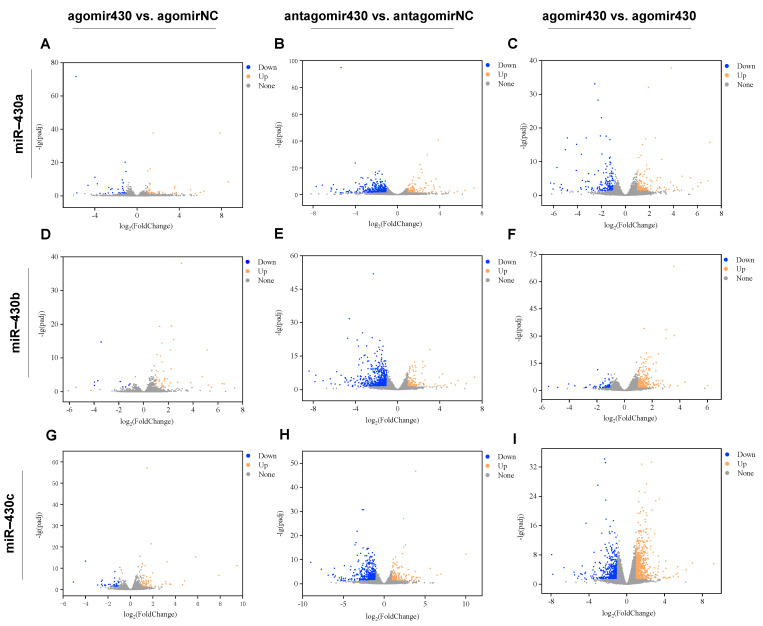
The statistics of DEGs identified in pairwise comparisons. (**A**) The agomir430a group compared with the agomirNC group; (**B**) The antagomir430a group compared with the antagomirNC group; (**C**) The agomir430a group compared with the antagomir430a group; (**D**) The agomir430b group compared with the agomirNC group; (**E**) The antagomir430b group compared with the antagomirNC group; (**F**) The agomir430b group compared with the antagomir430b group; (**G**) The agomir430c group compared with the agomirNC group; (**H**) The antagomir430c group compared with the antagomirNC group; (**I**) The agomir430c group compared with the antagomir430c group. *n* = 3 for each microinjection group.

Sequencing data quality of RNA-seq was exhibited in Table S1. The volcano plots showed that miR-430-injection significantly altered the transcriptome properties in *M. albus* (Figure 1). A total of 149 DEGs (agomir430a vs. agomirNC; 69 upregulated and 80 downregulated), 124 DEGs (agomir430b vs. agomirNC; 105 upregulated and 19 downregulated), 227 DEGs (agomir430c vs. agomirNC; 145 upregulated and 82 downregulated), 953 DEGs (antagomir430a vs. antagomirNC; 256 upregulated and 697 downregulated), 1577 DEGs (antagomir430b vs. antagomirNC; 369 upregulated and 1208 downregulated), 1122 DEGs (antagomir430c vs. antagomirNC; 301 upregulated and 821 downregulated), 827 DEGs (agomir430a vs. antagomir430a; 437 upregulated and 390 downregulated), 1141 DEGs (agomir430b vs. antagomir430b; 827 upregulated and 314 downregulated), and 3508 DEGs (agomir430c vs. antagomir430c; 1967 upregulated and 1541 downregulated) were identified in various treatment groups (Table S2). A total of 9, 58, and 36 DEGs were shared in both the miR-430a/b/c-overexpressing and -knockdown groups (Figure S3).

The potential functions and metabolic pathways of DEGs were further analyzed by using GO enrichment and KEGG pathways analyses. Based on sequence homology, the GO functional analysis showed that DEGs fell into the top 30 subcategories of three major categories: biological process, cellular component, and molecular function (Table S3). The details of GO functional analysis by pairwise comparisons among the eight treatment groups were listed in Supplementary GO terms.xlsx. In the biological process category, the most dominant subcategories were comprised of DNA rewinding and DNA strand renaturation, which was followed by metabolic process for the miR-430a-injected group. The lipid biosynthetic process and protein heterotrimerization were observed to be the most dominant subcategories for the miR-430b- and miR-430c-injected groups, respectively. In the cellular component category, nuclear replication fork and replication fork were the most predominant subcategories for the miR-430a-injected group. The perinuclear endoplasmic reticulum and tricarboxylic acid cycle enzyme complex were the most dominant subcategories for the miR-430b- and miR-430c-injected groups, respectively. In the molecular function category, DEGs were mainly clustered into the dominant subcategories of annealing helicase activity, 1-acylglycerol-3-phosphate O-acyltransferase activity, and oxidoreductase activity for the miR-430 (a, b, c) treatments, respectively. The coefficient matrix was shown in Figure S4, and the sample-to-sample distances were presented in Figure S5.

KEGG analysis was performed to investigate the functional classification and pathway enrichment of DEGs with a threshold of *p* < 0.05. In all miRNA-430 (a, b, c) treatments, steroidogenesis-related pathways such as the steroid biosynthesis pathway, steroid hormone biosynthesis pathway, MAPK signaling pathway, ErbB signaling pathway, Wnt signaling pathway, and GnRH signaling pathway were significantly enriched with DEGs. The results indicated that DEGs in agomir430a vs. agomirNC, antagomir430a vs. antagomirNC, and antagomir430a vs. agomir430a were significantly enriched in a total of 4, 19, and 13 pathways, respectively (Table S4). For miR-430b treatments, DEGs (agomir430b vs. agomirNC, antagomir430b vs. antagomirNC, and antagomir430b vs. agomir430b, respectively) were significantly enriched in 9, 25, and 27 pathways, and all of them included MAPK signaling, PPAR, ErbB signaling, Wnt signaling, and steroid hormone biosynthesis pathways. For miR-430c treatments, a total of 9, 17, and 49 pathways were significantly enriched with DEGs by pairwise comparisons (agomir430c vs. agomirNC, antagomir430c vs. antagomirNC, and antagomir430c vs. agomir430c, respectively), and these DEG enrichment pathways included MAPK, TGF-beta, and ErbB signaling pathways. Taken together, these results indicated that miR-430 micro-injection might significantly influence steroidogenesis-related pathways.

#### 2.2.3. Expression of Steroidogenesis-Related Genes in *M. albus*

**Figure 2:**
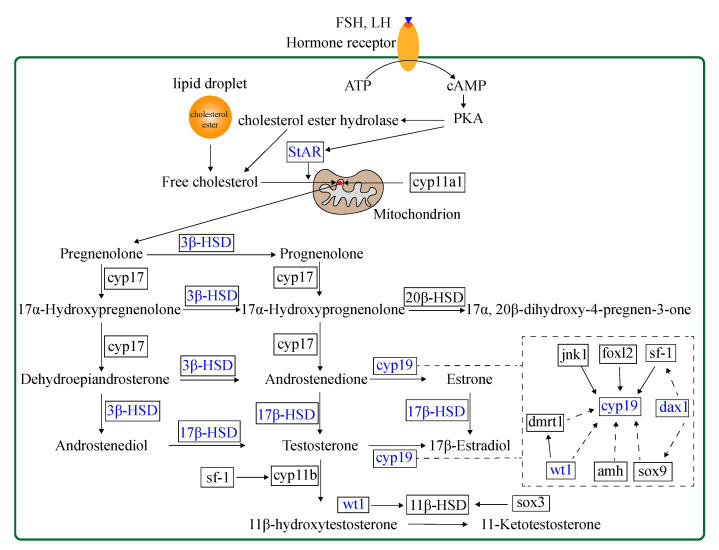
A schematic pathway of steroidogenesis and regulation of steroidogenic enzyme genes with its transcription factors in the gonads of teleost [22,32]. Words in blue represent genes that are upregulated or downregulated by miR-430 in rice field eel.

**Figure 3:**
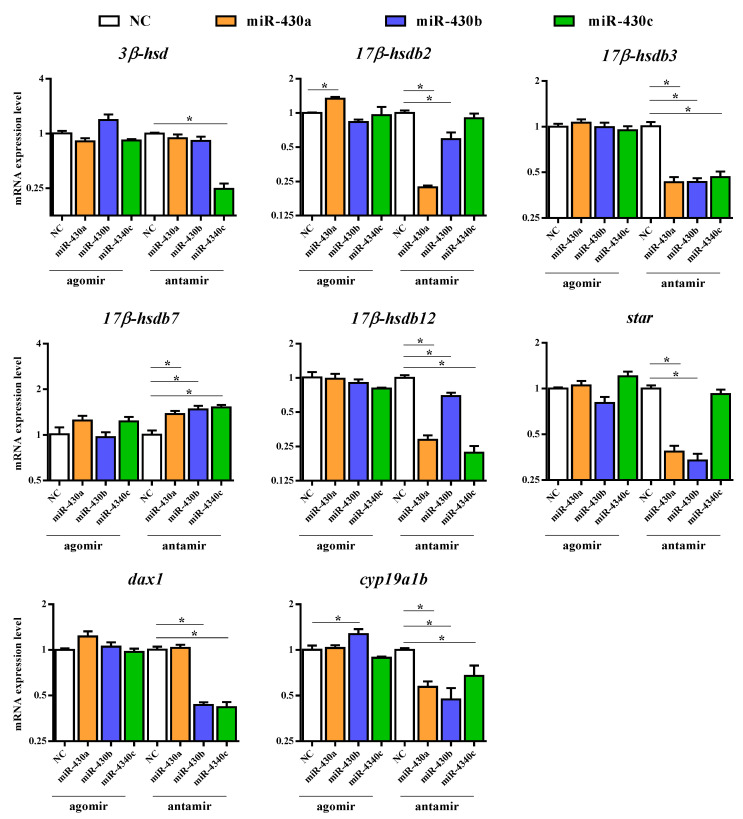
Candidate genes expression levels revealed by quantitative qPCR. The results of qPCR were performed by relative expression using *β-actin* and *ef1a* as the reference gene and measured by the method of optimized comparative Ct (2^-ΔΔCt^) value. Values are means ± SEM., *n* = 3. * *p* < 0.05.

To explore the potential effect of miR-430 on steroidogenesis, we investigated 30 relevant genes, including *vasa*, steroidogenic acute regulatory protein (*star*), nuclear orphan receptor (*dax1*), *cyp11b*, *cyp17*, 3-beta-hydroxysteroid dehydrogenase (*3β-hsd*), hydroxysteroid 17-beta dehydrogenase (*17β-hsd*), 20-beta-hydroxysteroid dehydrogenase (*20β-hsd*), hydroxysteroid 11-beta dehydrogenase (*11β-hsd*), *cyp19a1a*, *cyp19a1b*, *wt1*, *sf-1*, gonadal soma-derived factor (*gsdf*), SRY-box transcription factor 3 (*sox3*), *sox9*, *amh*, *dmrt1*, *foxl2*, forkhead box L3 (*foxl3*), c-Jun N-terminal kinase 1 (*jnk1*), androgen receptor (*ar*), estrogen receptor alpha (*erα*), estrogen receptor beta (*erβ*), and so on to validate RNA-Seq results by qPCR. The steroidogenesis-related pathway and genes regulated by transcription factors in the gonads of teleost are shown in Figure 2. The expression patterns of the 30 genes obtained by qPCR analysis were similar to those obtained by RNA-Seq, indicating that the results of the mRNA sequencing analysis were reliable and valid. Gene expression of *vasa*, *star*, *cyp11b*, *20β-hsd*, *11β-hsd*, *gsdf*, *sox3*, *sox9*, *amh*, *dmrt1*, *foxl2*, *foxl3, ar, erα*, and *erβ* were not significantly regulated in the agomir430- and antagomir430-treated groups compared with the corresponding NC (Figure S6), which is similar to that in RNA-Seq analysis (Table S7). Specifically, *17β-hsdb2* mRNA expression was significantly higher in the agomir430a group than in the agomirNC group (*p* < 0.05), and the similar observation was found in *cyp19a1b* gene expression in the agomir430b group (Figure 3). However, no significant difference in the expressions of other candidate genes was observed between the miRNA-overexpression treatment groups and the corresponding negative control. On the contrary, miRNA430 knockdown significantly declined the mRNA expression of *17β-hsdb3*, *17β-hsdb7*, *17β-hsdb12*, and *cyp19a1b* (*p* < 0.05) (Figure 3). In addition, mRNA expressions of *17β-hsdb2* and *star* were reduced in the miRNA430a knockdown and miRNA430b knockdown groups (*p* < 0.05), and mRNA expression of *dax1* was lower in the miR-430b knockdown and miRNA430c knockdown groups than in the corresponding miRNA NC group (*p* < 0.05). Decreased expression of *3β-hsd* was only detected in the miRNA430c knockdown group (*p* < 0.05) (Figure 3). Taken together, these results indicated that miR-430 microinjection could directly regulate steroidogenesis at the transcriptional level in larval *M. albus*.

### 2.3. Molecular Function Identification of miR-430 Family Genes in M. ablus

**Figure 4:**
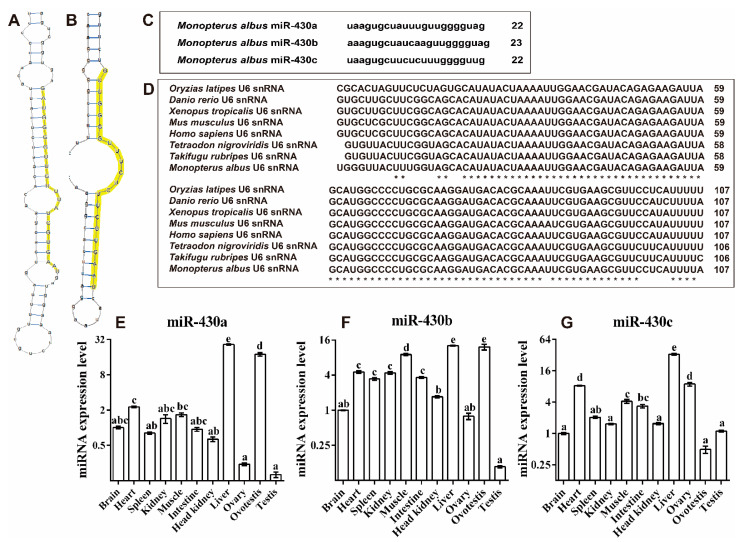
Identification of miR-430 family in rice field eel. Predicted secondary structures of miR-430a (**A**) and miR-430c (**B**) precursors in rice field eel. Representatives of each of two miR-430 groups are shown. Yellow shadings indicate mature regions and seed sequences, respectively. (**C**) miR-430 (a, b, c) sequences in rice field eel. (**D**) ClustalX alignment of the U6 snRNA from *M. albus* and other species. The star (*) represents the identical nucleotides. (**E**–**G**) The miRNA expression level of miR-430 in the brain, heart, spleen, kidney, muscle, intestine, liver, ovary, ovotestis, and testis. Data (mean ± SEM, *n* = 3) were normalized to the housekeeping gene (U6 snRNA). Bars that do not share a letter are significantly different among different tissues (*p* < 0.05).

In our previous studies, miR-430 family genes were identified (Figure 4C) [11]. To exploit the secondary structures of miR-430 gene family genes in *M. albus*, we searched genomic regions for the genes encoding miR-430. Secondary structures of miR-430 precursor sequences were predicted using RNAshapes software [33]. miR-430a and miR-430c were folded into a typical stem-loop structure with mature sequence in the 3′ end of the loop region, but miR-430b was not folded, which might be due to incomplete (fragmentary) genomic sequences of rice field eel (Figure 4A,B).

The highest expression level of miR-430a was detected in liver, followed by ovotestis and heart; it showed no significant differences among muscle, brain, kidney, intestine, spleen, and head kidney tissues, and the lowest expression level was detected in ovary and testis (Figure 4E). The highest expression level of miR-430b was observed in liver, followed by ovotestis and muscle; it exhibited no significant differences among heart, kidney, intestine, and spleen tissues, and the lowest expression was detected in head kidney, brain, ovary, and testis (Figure 4F). The highest expression level of miR-430c was also found in liver, followed by heart, ovary, muscle, intestine, spleen, and the lowest was found in kidney, with no differences among the tissues of head kidney, brain, testis, and ovotestis (Figure 4G). Taken together, these results revealed that miR-430a and miR-430b exhibited the highest expression in ovotestis among three gonads, and miR-430c displayed a higher expression in ovary than in ovotestis and testis.

### 2.4. Expressions of miR-430 Target Genes Cytochrome P450 Family 17(cyp17), Cytochrome P450 Family 19 Subfamily a (cyp19a1b), and Forkhead Box L2 (foxl2)

**Figure 5:**
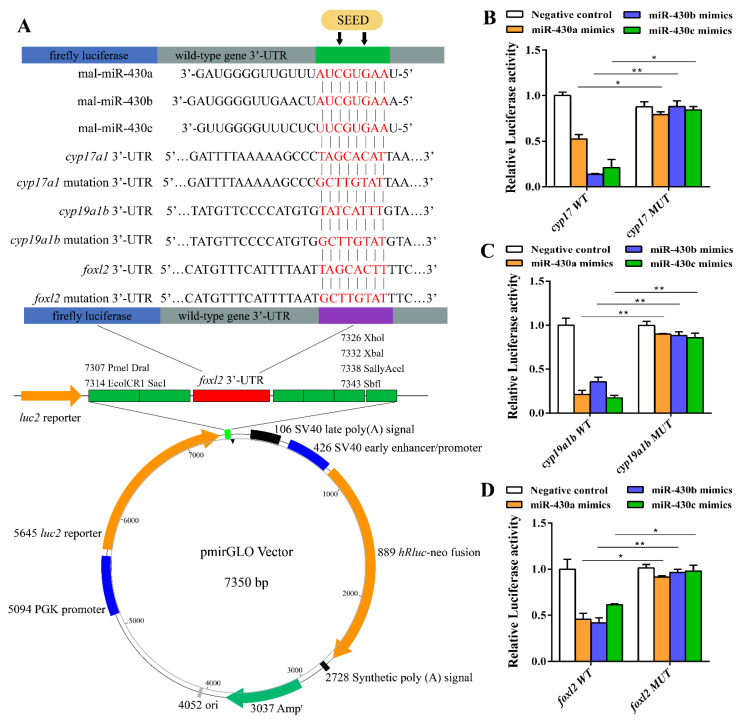
Schematic representation of the miR-430 target sequence within the 3′UTR of *cyp17*, *cyp19a1b*, and *foxl2* (**A**) and activities of the luciferase reporter gene linked to the 3′UTR of *cyp17* (**B**), *cyp19a1b* (**C**), and *foxl2* (**D**). The pmirGLO luciferase reporter plasmids with the wild-type or mutated 3′UTR sequences of *cyp17*, *cyp19a1b*, and *foxl2* were transiently transfected into HEK-29T cells along with 20 µM miR-430 mimics or negative control. Luciferase activities were measured after 24 h, and firefly luciferase was used for normalization. Values are means ± SEM., *n* = 3. * *p* < 0.05, ** *p* < 0.01. The experiment was repeated at least three times.

To further determine whether *cyp17*, *cyp19a1b*, and *foxl2* were regulated by miR-430s, we analyzed the 3′ UTR sequences of *cyp17*, *cyp19a1b*, and *foxl2* derived from NCBI by luciferase reporter assay. Alignment results of miR-430 sequences with the 3′ UTR sequences of *cyp17*, *cyp19a1b*, and *foxl2* indicated that miR-430 binding sites were detected in the 3′ UTR of these genes (Figure 5A). The miR-430 mimics had a marked repressive effect on luciferase reporter activities of wild-type 3′ UTR of *cyp17*, *cyp19a1b*, and *foxl2* (Figure 5B–D). In addition, mutation of the miR-430 binding sites in 3′ UTR of *cyp17*, *cyp19a1b*, and *foxl2* completely abolished the inhibition of luciferase reporter activities by miR-430, validating the regulatory effects of the miR-430s on the expression of *cyp17*, *cyp19a1b*, and *foxl2* (Figure 5). Taken together, our results indicated that miR-430s could suppress the expressions of *cyp17*, *cyp19a1b*, and *foxl2* by binding to the target sites of their 3′ UTR.

## 3. Discussion

Although previous studies have found the important roles of miR-430s in morphogenesis and fish development, the regulatory effect of miRNA-430s on steroidogenic alteration has scarcely been reported in fish. In the present study, a deep sequencing of mRNA from hatched *M. albus* larvae after microinjecting embryos with miR-430 mimics and inhibitors was performed to study the relationship between miR-430s and steroidogenesis. Furthermore, the molecular characteristics, tissue distribution, and steroidogenisis-related target genes of miR-430s were analyzed in larval rice field eel.

Steroidogenesis can stimulate gonads to develop and differentiate into testis or ovary [34,35]. Our previous study has identified numerous miRNAs in *M. albus* and found the miR-430 family to be involved in the sexual transformation [11]. Considering that sexual transformation was related to steroidogenesis, we speculated that miR-430s might play a role in regulating steroidogenesis-related gene expression in fish. In the present study, a total of >149 DEGs were identified through pairwise comparisons (agomir430s vs. agomirNC, antagomir430s vs. antagomirNC, agomir430s vs. antagomir430s) of eight treatments. GO and KEGG analyses indicated that miR-430s were involved in steroidogenesis pathways including the regulation of the G protein-coupled receptor signaling pathway, steroid hormone biosynthesis pathway, estrogen signaling pathway, PPAR signaling pathways, PI3K–Akt signaling pathways, Wnt signaling pathways, and erbB signaling pathways. Notably, the signaling crosstalk among the PI3K–Akt pathway and steroid hormone biosynthesis pathway was activated by the binding of FSH to specific cell-surface G protein-coupled receptors (GPCRs), thus facilitating the differentiation and development of ovary [36]. In the present study, 24 DEGs enriched the regulation of the G protein-coupled receptor signaling pathway after miR-430a knockdown (antamir430a vs. antamirNC). In addition, DEGs by knockdown of miR-430b were enriched in steroid hormone biosynthesis, which revealed that miR-430s could affect steroidogenesis-related genes in rice fields [37]. Moreover, miR-430 (a, b) knockdown significantly downregulated *star*, limiting the rate of steroidogenesis [38]. PPAR, Wnt, and PI3K-Akt signaling pathways have been reported to be involved in intra-ovarian pathways, which is also important for regulating ovarian function [39]. Altogether, these results suggested that miR-430s could affect steroidogenesis and sex differentiation by altering the expression levels of genes involved in G protein-coupled receptor, Wnt, PPAR, PI3K–Akt signaling pathways in larval rice field eel.

Based on above findings, a large number of functional DEGs involved in steroidogenesis and sex differentiation were also identified, revealing the vital roles of miR-430s in the reproduction of fish. In the present study, the overexpression of miR-430 (a, rather than b or c) significantly upregulated *17β-hsdb2* expression, which indicated that miR-430a more effectively regulated *17β-hsdb2* than miR-430b and miR-430c. Interestingly, of the 30 genes selected for qPCR validation, some other candidate genes except *17β-hsdb2* were not significantly expressed after the overexpression or knockdown of miR-430s at early embryonic development, although they were confirmed to be highly expressed in either ovary or testis of *M. albus* [10,11]. In addition, the microinjection of miR-430s did not significantly alter expressions of steroidogenic genes, which might be attributed to the high level of miR-430s in *M. albus* embryo itself. Previous studies have reported a high expression of miR-430s during the early stages of larval fish [24,40], which indicated the pivotal role of miR-430 in fish development. Moreover, the knockdown of miR-430s significantly decreased the expressions of *17β-hsdb3*, *17β-hsdb7*, *17β-hsdb12*, and *cyp19a1b*. Notably, 17B-HSD and CYP19 are the key regulatory enzymes involved in steroidogenesis [41]. The reduced gene expressions induced by miR-430s knockdown revealed that miR-430s might be essential for fish development and contribute to steroidogenesis. Altogether, these findings confirmed that miR-430 could directly mediate steroidogenesis at the transcriptional level in rice field eel.

It is well-known that the miRNA/mRNA relationship in animals is complicated [42]. In our previous study, the bioinformation analysis of the complete 3′UTR of potential genes revealed that *cyp17*, *cyp19a1b*, and *foxl2* contained putative miR-430 binding sites; thus, it could be concluded that miR-430s could regulate *cyp17*, *cyp19a1b*, and *foxl2* expression by targeting their 3′UTRs [11]. CYP19 (P450arom), the key steroidogenic enzyme responsible for the conversion of androgens into estrogens, was translated by the *cyp19* genes, including *cyp19a1a* (ovarian aromatase) and *cyp19a1b* (brain aromatase) [12,43,44]. *Foxl2* has been reported as a putative transcription factor to facilitate the maturation of female vertebrate gonad by activating *cyp19* translation in the early development of fish [10]. To validate whether these predicted target genes were regulated by miR-430s, we ligated their 3′UTRs to the Renilla luciferase (RL) reporter and analyzed the reporter’s activities, and we found that all these target genes (*cyp17*, *cyp19a1b*, and *foxl2*) were suppressed by miR-430s. However, the knockdown of miR-430s could not significantly increase the expression of *cyp19a1b*, indicating that miR-430s were not the single repressor for *cyp19a1b* expression. One previous study showed that *foxl2* regulated *cyp19a1b* expression, and that miR-430 was a direct repressor of *foxl2* expression [11]. However, our data indicated that *foxl2* gene expression was not affected by miR-430 overexpression or knockdown in *M. albus*. Such a difference might be attributed to the possibility that miR-430s might directly/indirectly alter the *cyp19a1b* gene expression by affecting the mRNA expression of *foxl2*. In tilapia, miR-338/*cyp17a2*, miR-200a/*cyp17a2*, miR-7977/*foxl2*, and miR-96/*hsd3b* were involved in steroid synthesis, suggesting a complex miRNA regulatory network during sex differentiation [22,32]. All these results strongly suggested a role of miR-430s in regulating the biosynthesis of steroid hormones by directly targeting *cyp19a1b* or indirectly targeting *cyp19a1b* via *foxl2* during the early development of rice field eel.

To further investigate the potential roles of miR-430s in regulating the steroidogenesis of rice field eel, we conducted a miR-430 bioinformation analysis and found that in *M. albus*, various miR-430 precursors possessed the identical mature sequences containing the homologous seed sequences, indicating that various miR-430s might share the identical evolutionary origin [25]. Notably, miR-430 contained more than 70 copies in zebrafish, which facilitated the early generation of the miR-430 transcript during embryogenesis [27]. Zebrafish and medaka have been reported to possess two large categories of miR-430 genes on chromosome 4, implying that the common ancestor of teleosts might possess multiple tandemly clustered miR-430 genes [27,28]. In contrast, the genomic composition of the miR-430 family was not found in *M. albus*, which might be due to the absence of integral genome. More studies are needed to uncover the miR-430 candidate genes and their chromosomal organization. In fish, miRNA distribution in the reproductive axis (including brain, pituitary, and gonad) has been reported in yellow catfish [45], Nile tilapia [32,46,47], and rice field eel [11]. This study examined the miR-430s distribution in different tissues and revealed that miR-430s were extensively expressed in various tissues of rice field eel. In addition, the different expressions of miR-430s in three gonads (ovary, ovotestis, and testis) further indicated the importance of miR-430s in fish steroidogenesis. Particularly, high expressions of miR-430a and miR-430b in ovotestis confirmed that miR-430 (a and b) facilitated the sex reversal from female to male via intersex in *M. albus* [11]. However, the highest expression of miR-430c was observed in ovary among three gonads, implying that three miR-430s played different roles in the sex reversal of rice field eel. Taken together, miR-430 tissue distribution in rice field eel suggested the distinct functions of different isoforms of miR-430 in steroidogenesis.

## 4. Materials and Methods

### 4.1. Ethical Statement

All the experiments on animals and cells were conducted under the guidance of Huazhong Agricultural University (HZAU) for the care and use of laboratory animals and were approved by the Ethical Committee of HZAU. The approval number is HZAUFI-2019-020 (Approval date: 7 May 2019).

### 4.2. Chemical Reagents

Agomir and antagomir of miR-430s and negative control were purchased from Shanghai GenePharma Co., Ltd. (Shanghai, China). The sequences of three miR-430s used for microinjection were 5′-uaagugcuauuuguugggguag-3′ (miR-430a), 5′-aaagugcuaucaaguugggguag-3′ (miR-430b), and 5′-uaagugcuucucuuugggguug-3′ (miR-430c), respectively. MS-222 and L-glutamine were obtained from Amresco (Solon, OH, USA). Dulbecco’s modified Eagle’s medium (DMEM, high glucose) and fetal bovine serum (FBS) were obtained from Gibco/Invitrogen (Paisley, UK). Penicillin and streptomycin were purchased from Sigma-Aldrich (St. Louis, MO, USA). Lipofectamine2000 and pmirGLO vector were obtained from Invitrogen (Carlsbad, CA, USA). A ClonExpress™ II One Step Cloning Kit was purchased from Vazyme (Piscataway, NJ, USA). A Dual-Luciferase Report assay was obtained from Promega (Madison, WI, USA). A TaKaRa PrimeSTAR^®^ HS DNA Polymerase kit was purchased from TaKaRa (Tokyo, Japan).

### 4.3. Microinjection of miRNA Mimics and Inhibitors

Fertilized eggs were obtained from the Aquatic Germplasm Resources Preservation and Varieties Breeding Center of the Yangtze River Fisheries Research Institute, Chinese Academy of Fishery Sciences. One-cell stage embryos were used for microinjection. Embryos of the rice field eel were injected with miRNA-overexpressing mimics (agomirNC (negative control), agomir430a, agomir430b, and agomir430c) or miRNA-knockdown inhibitors (antagomirNC (negative control), antagomir430a, antagomir430b, and antagomir430c) at 3.3 μmol/μL in triplicate. One μL miRNA was injected into 500 embryos of rice field eel. Subsequently, these embryos were placed on net sheets surrounded by plastic frames on water surface at water temperatures of 28–31 ℃, as previously reported [48]. Unfertilized or seriously abnormal embryos were discarded. Developmental stages were expressed as hours and days after fertilization. The experiment continued for 1 week, when the hatching fish were exhibited as Figure S1l. All fish from each group were frozen in liquid nitrogen immediately and stored at −80 °C for total RNA extraction.

### 4.4. Determination of miRNA Expression by qRT-PCR

Nine fish from each group were randomly selected to detect the miR-430 expression after microinjection. The total RNAs were extracted using TRIzol regent (Takara, Tokyo, Japan) according to the manufacturer’s instructions. mRNA reverse transcriptions were performed with equal amounts of total RNA (1 µg) from each sample as a template with Quantitect Reverse Transcription Kit (Takara) following the manufacturer’s protocol. The stem-loop qRT-PCR of microRNAs was performed. Oligo dT/Random primers were replaced with miR-430s stem-loop RT primers in a TAKARA Reverse Transcription Kit (Table S5). The qRT-PCR was performed with the SYBR Premix Ex TaqTM II kit (Takara) on a Chromo 4 Real-Time Detection System (MJ Research, Hercules, CA, USA) following the manufacturer’s protocols in the previously reported method [10]. The gene-specific primers for each gene are listed in Table S5. The relative expression levels of miR-430s were analyzed using the comparative ΔΔCt method, and they were normalized with U6 (Figure 5D). All experiments were performed in triplicate.

### 4.5. RNA-Seq

Twenty-four sequencing samples (3 total RNAs from each group, 8 miRNA microinjection groups) with high RNA quality were selected based on the detection of miR-430 expression levels (Table S6), and were sent to Annoroad Gene Technology Co. Ltd. (Beijing, China). RNA integrity and concentration were assessed using the RNA Nano 6000 Assay Kit of the Bioanalyzer 2100 system (Agilent Technologies, CA, USA). The samples with RNA integrity number (RIN) ≥7 were subjected to the subsequent analysis. Sequence libraries were constructed by purifying and fragmenting the mRNA. The first-strand cDNA was synthesized. Based on this first-strand cDNA, the second-strand cDNA was synthesized with its 3′ ends adenylated, and its adapters were ligated to enrich the DNA fragments. The resultant DNA fragment products were purified, and then, the DNA libraries were validated. Subsequently, these libraries were sequenced on an Illumina platform (HiSeqTM 2500; Illumina Inc., San Diego, CA, USA), and 150 bp paired-end reads were generated. 

The bioinformatics analysis was as follows: (1) Quality control and mapping. Raw data (raw reads with Fastq format) were processed with the NGS QC Toolkit (OMICTools, Le-Petit-Quévilly, France). All reads have been submitted to NCBI, and the accession number is SAMN19709588-SAMN19709635. The low-quality, adaptor-polluted, and high content of unknown base (N) (base quality score < Q30, where Q30 = quality value (>30)/total base) reads were filtered by GT proprietary tools to obtain clean reads [49]. The clean reads were mapped to a reference genome (ID: *Monopterus albus*) using HISAT2. (2) Gene-level quantification, DEG analysis, cluster analysis, GO- and KEGG enrichment analysis. The fragments per kilobase of transcript per million mapped reads (FPKM) value of each gene was calculated using cufflinks. Read counts for each gene were obtained with htseq-count. Normalization of reads was conducted by DESeq2 [50]. Independent filtering in DESeq2 was conducted to filter out genes with low read counts. The DEGs were identified using the DESeq2 [51]. Gene ontology (GO) enrichment and Kyoto encyclopedia of genes and genomes (KEGG) pathway analyses of the DEGs were performed with R package based on a hypergeometric distribution. (3) Gene structure extension and novel transcript identification. Clean reads were reassembled using cufflinks. Gene structure extension and novel transcript identification were performed by mapping the reference genome with known annotated genes using cuffcompare.

DEGs were identified by comparing agomir430 with the agomirNC or antagomir430 with antagomirNC. Genes found by DESeq with fold-change (FC) > 1.5, *p* < 0.05 and FDR-adjusted *p* value < 0.05 were designated as DEGs. Upregulated DEGs (Up_diff) or downregulated DEGs (down_diff) were classified based on FPKM value. The DEGs were subjected to GO and KEGG analyses to determine their possible functions and enriched pathways using the GO annotation system and DAVID online tool, respectively.

### 4.6. RNA-Seq Result Validation through Quantitative Real-Time PCR

Thirty genes were selected for the validation of RNA-Seq results by qPCR following the manufacturer’s instructions. The specific primers of each gene are listed in Table S5. The relative expression levels of these 30 genes were analyzed using the comparative ΔΔCt method, and the gene expression levels were normalized with *β-actin* and *ef1a*. All experiments were performed in triplicates.

### 4.7. miR-430 Molecular Structure Analysis and Its Target Gene Verification

#### 4.7.1. Prediction of Secondary Structure of microRNA Precursors in *M. albus*

Sequences of miR-430s from various animal species were obtained in our previous studies [11]. Specifically, zebrafish miR-430 precursor sequences registered in miRBase were blasted against the rice field eel genome. The identified sequences of miR-430 precursors of the rice field eel were aligned using ClustalX software [52,53]. The overall structures (5′-star-loop-mature-3′) were investigated to ensure the perfect conservation of seed sequences. Secondary structures of candidate miR-430s were predicted using RNAshapes software [33].

#### 4.7.2. Expression Levels of miR-430 in Various Tissues during Different Developmental Stages

Approximately 40 rice field eels were purchased from Baishazhou Agricultural and Sideline Products Market. The rice field eels were raised temporarily in a large water tank (4 × 2 × 1.5 m) at 23.3 °C ± 0.5 °C with a 14 h light/10 h dark photoperiod for 1 week. On the sampling day, they were anesthetized (MS-222 at 500 mg/L) and sacrificed by decapitation. Brain, heart, spleen, kidney, muscle, intestine, liver, and part of the gonad were collected for RNA extraction, and the remaining part of the gonad was stored in 4% poly-formaldehyde fixative for histological analysis to determine the sexual stage. Total RNA was extracted, and qRT PCR was performed according to the manufacturer’s protocol with slight modifications, as previously reported [10].

#### 4.7.3. Verification of Target Genes of miR-430s

Human embryonic kidney (HEK) 293T cells were cultured in DMEM containing 10% FBS, 100 U/mL penicillin, 100 mg/mL streptomycin, and 250 ng/mL amphotericin B (Invitrogen) at 5% CO_2_ and 37 °C. The 300–500 bp wild-type DNA fragments of the 3′UTR of *cyp17*, *cyp19a1b*, and *foxl2* mRNA containing the putative binding sites with miR-430 were synthesized by PCR. Then, the obtained fragments were subcloned into the Renilla luciferase coding region downstream in the pmirGLO vector (Promega) through the SacI and XhoI sites, and these fragments were ligated to this vector using a ClonExpress™ II One Step Cloning Kit (Vazyme). The 8 bases complementary to the miR-430 seed sequence were mutated into GCTTGTAT to obtain recombinant plasmids by overlap-PCR, and these mutated recombinant plasmids were validated by sequencing in Tsingke (Wuhan, China). The PCR reactions were performed using TaKaRa PrimeSTAR^®^ HS DNA Polymerase kit (TaKaRa). HEK-293T cells were cultured at 1 × 105 cells/well on 24-well plates and co-transfected with pmirGLO-*foxl2*-3′UTR (pmir-*foxl2*, 0.2 µg), pmirGLO-mut*foxl2*-3′UTR (pmir-*foxl2*-mut, 0.2 µg), empty vector plasmid (0.2 µg), and miR-430 mimics (20 pmol) or miR-430 negative control (20 pmol), respectively, by using Lipofectamine2000 (2 µL) kit. Cells were harvested at hour 48 after transfection.

Firefly and Renilla luciferase activities were analyzed using the Dual-Luciferase Report assay (Promega) according to the manufacturer’s instructions. Luciferase activity values were obtained using a Turner Biosystems 20/20n single-tube luminometer. Renilla luciferase activity was normalized to the firefly luciferase activity, and then, the obtained ratio was further normalized to the control constructs. At least four independent cotransfections under each condition were averaged. All experiments were performed in triplicates, and the data were measured at least three times.

### 4.8. Statistical Analyses

Data (raw reads, clean reads, valid base ratio, Q30 percentage, GC ratio, and gene expression) were expressed as mean ± standard error of mean (SEM). In clustering analysis, heatmap plots were created based on Log10 transformed relative intensities of DEGs among the three groups. In GO enrichment and KEGG pathway analyses, *p* < 0.05 was designated as a significant enrichment of DEGs. Statistical analysis was performed with SPSS 19.0 software (SPSS, Michigan Avenue, Chicago, IL, USA).

## 5. Conclusions

miR-430s can promote the steroidogenesis and the biosynthesis of steroid hormones by directly targeting *cyp19a1b* or indirectly targeting *cyp19a1b* via *foxl2*. Our data reveal the slight differences in molecular characteristics and potential physiological functions of miR-430s in rice field eel, which will be useful for further studies of steroidogenesis and sex reversal in fish. Overall, this study confirms the roles of miR-430s in regulating steroidogenesis and provides novel insights into the molecular mechanisms of endocrine, sex reversal, and reproduction in fish.

## Data Availability

Not applicable.

## Supplementary Materials

The following are available online at https://www.mdpi.com/article/10.3390/ijms22136994/s1. Table S1 Sequencing data quality. Table S2 Statistical result of DEGs (up and down). Table S3 Significant GO enrichment among the DEGs. Table S4 Significant KEGG enrichment among the DEGs. Table S5 Primers used for real-time PCR analysis. Table S6 RNA sample purity and integrity. Figure S1: The statistics of DEGs identified in pairwise comparisons. Figure S2: A Venn diagram showing the number of significantly differential expressed genes identified by 73 pairwise comparisons of different groups. Figure S3: Heatmap of the coefficient matrix for the species-abundance clustering image. Figure S4: Cluster dendrogram of the sample-to-sample distances for the species-abundance clustering 83 image.
