# Supplementary material for: Integrated Analysis of miR-430 on Steroidogenesis-Related Gene Expression of Larval Rice Field Eel Monopterus albus
Source: Int J Mol Sci. 2021 Jun 29;22(13):6994. doi: 10.3390/ijms22136994 (PMC8269179; doi:10.3390/ijms22136994)
Supplement: Supplementary file 1 [file ijms-22-06994-s001.zip › ijms-1230655-SI.pdf]

Table S1 Sequencing data quality.

Clean reads were obtained by removing low-quality reads and those containing adapter and poly-N from the raw data. Q30 = quality value (>30)/total base.

Sample	raw reads	clean reads	valid bases (%)	Q30 (%)	Mapping rate (%)
agomirNC	46774609±2035511	45766630±1951401	97.85±0.11	94.51±0.2	92.55±0.26
agomir430a	46086528±1412475	45131700±1344490	97.93±0.11	94.28±0.06	92.2±0.05
agomir430b	47978387±792476	46796464±719636	97.54±0.16	93.73±0.69	92.35±0.24
agomir430c	46906758±446238	45736631±514893	97.51±0.2	94.3±0.18	92.4±0.31
antagomirNC	46159767±988096	45032195±840140	97.56±0.58	94.35±0.15	92.1±0.19
antagomir430a	47575397±964445	46390373±1028522	97.51±0.19	94.37±0.16	91.73±0.36
antagomir430b	46688399±1218920	45415640±1195089	97.27±0.09	94.49±0.06	91.84±0.16
antagomir430c	46589309±1375614	45249100±1235378	97.13±0.41	94.36±0.23	92.01±0.34

Table S2 Statistical result of DEGs (up and down).

When we compared T11 with the C1, 149 DEGs (including 69 upregulated and 80 downregulated) were identified. C1, agomirNC; C2, antagomirNC; T11, agomir430a; T21, antagomir430a; T12, agomir430b; T22, antagomir430b; T13, agomir430c; T23, antagomir430c. n=3 for each microinjection group.

Control	Case	Up_diff	Down_diff	Total_diff
C1	T11	69	80	149
C1	T12	105	19	124
C1	T13	145	82	227
C2	C1	977	1967	2944
C2	T21	256	697	953
C2	T22	369	1208	1577
C2	T23	301	821	1122
T21	T11	437	390	827
T22	T12	827	314	1141
T23	T13	1967	1541	3508

Table S3 Top 10 significant GO enrichment among the DEGs.

Using q value to determine the significant value of the collected GO clusters, 0.01 represents significant significance. n=3 for each microinjection group.

Ontology	ID	Description	qvalue	Count
	**agomir430a vs. agomirNC**			
biological process	GO:0036292	DNA rewinding	9.07E-24	12
	GO:0000733	DNA strand renaturation	8.06E-21	12
	GO:0048478	replication fork protection	5.42E-20	12
	GO:0031297	replication fork processing	3.61E-17	12
	GO:2000104	negative regulation of DNA-dependent DNA replication	1.41E-16	12
	GO:0008156	negative regulation of DNA replication	4.74E-16	12
	GO:0045005	DNA-dependent DNA replication maintenance of fidelity	9.45E-16	12
	GO:0090329	regulation of DNA-dependent DNA replication	3.79E-15	12
	GO:0032392	DNA geometric change	9.35E-15	12
	GO:0045910	negative regulation of DNA recombination	3.86E-14	12
molecular function	GO:0036310	annealing helicase activity	1.83E-22	12
	GO:0070530	K63-linked polyubiquitin modification-dependent protein binding	1.81E-21	12
	GO:0097617	annealing activity	1.22E-20	12
	GO:0004520	endodeoxyribonuclease activity	3.11E-19	12
	GO:0004536	deoxyribonuclease activity	1.92E-17	12
	GO:0031593	polyubiquitin modification-dependent protein binding	2.65E-17	12
	GO:0004519	endonuclease activity	1.13E-16	14
	GO:0004518	nuclease activity	2.45E-14	14
	GO:0140097	catalytic activity, acting on DNA	3.10E-13	14
	GO:0140030	modification-dependent protein binding	2.70E-11	12
cellular component	GO:0043596	nuclear replication fork	1.75E-15	12
	GO:0005657	replication fork	9.15E-13	12
	GO:0005865	striated muscle thin filament	2.99E-07	6
	GO:0036379	myofilament	2.99E-07	6
	GO:0000228	nuclear chromosome	2.49E-05	12
	GO:0005694	chromosome	0.00015	14
	GO:0030018	Z disc	0.00015	6
	GO:0031674	I band	0.000208	6
	GO:0030017	sarcomere	0.002455	6
	GO:0030016	myofibril	0.00587	6

Ontology	ID	Description	qvalue	Count
	**agomir430b vs. agomirNC**			
biological process	GO:0008610	lipid biosynthetic process	2.59E-11	26
	GO:0006699	bile acid biosynthetic process	2.59E-11	9

	GO:0008206	bile acid metabolic process	2.79E-10	9
	GO:0015850	organic hydroxy compound transport	7.47E-10	16
	GO:0015918	sterol transport	3.94E-09	10
	GO:0019432	triglyceride biosynthetic process	5.43E-08	9
	GO:0072330	monocarboxylic acid biosynthetic process	5.43E-08	14
	GO:0046460	neutral lipid biosynthetic process	5.43E-08	9
	GO:0046463	acylglycerol biosynthetic process	5.43E-08	9
	GO:0016053	organic acid biosynthetic process	1.91E-07	15
molecular function	GO:0003841	1-acylglycerol-3-phosphate O-acyltransferase activity	3.86E-12	10
	GO:0016411	acylglycerol O-acyltransferase activity	3.86E-12	10
	GO:0004366	glycerol-3-phosphate O-acyltransferase activity	3.86E-12	8
	GO:0042171	lysophosphatidic acid acyltransferase activity	3.86E-12	10
	GO:0071617	lysophospholipid acyltransferase activity	3.86E-12	10
	GO:0015248	sterol transporter activity	5.78E-12	9
	GO:0008374	O-acyltransferase activity	4.51E-11	10
	GO:0016747	transferase activity, transferring acyl groups other than amino-acyl groups	2.53E-09	14
	GO:0016746	transferase activity, transferring acyl groups	2.79E-09	14
	GO:0005319	lipid transporter activity	4.11E-07	10
cellular component	GO:0097038	perinuclear endoplasmic reticulum	2.09E-14	9
	GO:0045239	tricarboxylic acid cycle enzyme complex	2.95E-06	5
	GO:0005789	endoplasmic reticulum membrane	1.90E-05	17
	GO:0098827	endoplasmic reticulum subcompartment	1.90E-05	17
	GO:0042175	nuclear outer membrane-endoplasmic reticulum membrane network	1.90E-05	17
	GO:0031965	nuclear membrane	7.81E-05	10
	GO:0005635	nuclear envelope	0.000297952	11
	GO:1990204	oxidoreductase complex	0.005057216	5
**Ontology**	**ID**	**Description**	**qvalue**	**Count**
	**agomir430c vs. agomirNC**			
biological process	GO:0070208	protein heterotrimerization	6.19E-08	6
	GO:0070206	protein trimerization	5.33E-06	6
	GO:0045333	cellular respiration	9.06E-06	9
	GO:0006103	2-oxoglutarate metabolic process	9.06E-06	5
	GO:0022028	tangential migration from the subventricular zone to the olfactory bulb	9.06E-06	5
	GO:0021859	pyramidal neuron differentiation	1.00E-05	5
	GO:0021860	pyramidal neuron development	1.00E-05	5
	GO:0051291	protein heterooligomerization	1.53E-05	9
	GO:0006099	tricarboxylic acid cycle	1.53E-05	5

	GO:0021772	olfactory bulb development	1.53E-05	6
molecular function	GO:0016491	oxidoreductase activity	0.000102346	13
	GO:0016620	oxidoreductase activity, acting on the aldehyde or oxo group of donors, NAD or NADP as acceptor	0.000102346	5
	GO:0016903	oxidoreductase activity, acting on the aldehyde or oxo group of donors	0.000134709	5
	GO:0019843	rRNA binding	0.003567774	4
	GO:0019842	vitamin binding	0.003567774	5
	GO:0051087	chaperone binding	0.003873008	5
	GO:0005506	iron ion binding	0.004767512	4
	GO:0031072	heat shock protein binding	0.005473835	5
	GO:1901681	sulfur compound binding	0.006939268	6
cellular component	GO:0045239	tricarboxylic acid cycle enzyme complex	1.72E-06	5
	GO:1990204	oxidoreductase complex	1.72E-06	8
	GO:0030312	external encapsulating structure	0.000566564	8
	GO:0031012	extracellular matrix	0.000566564	8
	GO:0042383	sarcolemma	0.000974441	6
	GO:0009295	nucleoid	0.001066259	4
	GO:0042645	mitochondrial nucleoid	0.001066259	4
	GO:0005759	mitochondrial matrix	0.001163166	9
	GO:0005788	endoplasmic reticulum lumen	0.001228406	7
	GO:0045171	intercellular bridge	0.001669443	4

**Ontology**	**ID**	**Description**	**qvalue**	**Count**
	**antagomir430a vs. antagomirNC**			
biological process	GO:0016056	rhodopsin mediated signaling pathway	5.75E-08	10
	GO:0008277	regulation of G protein-coupled receptor signaling pathway	5.75E-08	24
	GO:0022400	regulation of rhodopsin mediated signaling pathway	6.01E-08	9
	GO:0010612	regulation of cardiac muscle adaptation	6.72E-08	8
	GO:1903242	regulation of cardiac muscle hypertrophy in response to stress	6.72E-08	8
	GO:0090343	positive regulation of cell aging	1.99E-07	8
	GO:0007602	phototransduction	4.06E-07	11
	GO:0007603	phototransduction, visible light	5.62E-07	10
	GO:0007084	mitotic nuclear envelope reassembly	8.72E-07	8
	GO:0101024	nuclear membrane organization involved in mitotic nuclear division	8.72E-07	8
molecular function	GO:0008157	protein phosphatase 1 binding	2.39E-11	12
	GO:0004712	protein serine/threonine/tyrosine kinase activity	4.63E-07	10
	GO:0047555	3',5'-cyclic-GMP phosphodiesterase activity	2.60E-06	7

	GO:0005201	extracellular matrix structural constituent	4.39E-06	8
	GO:0099583	neurotransmitter receptor activity involved in regulation of postsynaptic cytosolic calcium ion concentration	7.61E-06	7
	GO:0003916	DNA topoisomerase activity	1.72E-05	6
	GO:0005221	intracellular cyclic nucleotide activated cation channel activity	1.72E-05	6
	GO:0043855	cyclic nucleotide-gated ion channel activity	1.72E-05	6
	GO:0015459	potassium channel regulator activity	1.98E-05	10
	GO:0004857	enzyme inhibitor activity	0.000132121	22
cellular component	GO:0005882	intermediate filament	1.16E-12	16
	GO:0045111	intermediate filament cytoskeleton	7.05E-09	16
	GO:0005615	extracellular space	1.38E-08	47
	GO:0005652	nuclear lamina	2.52E-08	8
	GO:0005788	endoplasmic reticulum lumen	3.92E-08	22
	GO:0097381	photoreceptor disc membrane	7.58E-08	9
	GO:0042622	photoreceptor outer segment membrane	6.01E-07	10
	GO:0060170	ciliary membrane	6.82E-05	12
	GO:0005834	heterotrimeric G-protein complex	0.000119809	6
	GO:1905360	GTPase complex	0.000119809	6
**Ontology**	**ID**	**Description**	**qvalue**	**Count**
	**antagomir430b vs. antagomirNC**			
biological process	GO:0036292	DNA rewinding	1.63E-12	12
	GO:0098884	postsynaptic neurotransmitter receptor internalization	1.63E-12	12
	GO:0099590	neurotransmitter receptor internalization	1.63E-12	12
	GO:0140239	postsynaptic endocytosis	1.63E-12	12
	GO:1904978	regulation of endosome organization	1.63E-12	12
	GO:1904980	positive regulation of endosome organization	1.63E-12	12
	GO:1903423	positive regulation of synaptic vesicle recycling	8.01E-12	13
	GO:1903421	regulation of synaptic vesicle recycling	1.43E-09	13
	GO:0000733	DNA strand renaturation	1.56E-09	12
	GO:0048478	replication fork protection	1.28E-08	12
molecular function	GO:1990175	EH domain binding	1.83E-12	12
	GO:0043812	phosphatidylinositol-4-phosphate phosphatase activity	1.12E-11	12
	GO:0036310	annealing helicase activity	4.93E-11	12
	GO:0004520	endodeoxyribonuclease activity	3.53E-10	14
	GO:0070530	K63-linked polyubiquitin modification-dependent protein binding	5.25E-10	12
	GO:0004536	deoxyribonuclease activity	2.47E-09	15
	GO:0043813	phosphatidylinositol-3,5-bisphosphate 5-	2.97E-09	12

		phosphatase activity		
	GO:0097617	annealing activity	2.97E-09	12
	GO:0140097	catalytic activity, acting on DNA	5.41E-09	27
	GO:0052658	inositol-1,4,5-trisphosphate 5-phosphatase activity	1.43E-08	12
cellular component	GO:0030132	clathrin coat of coated pit	4.04E-13	14
	GO:0005882	intermediate filament	1.14E-11	18
	GO:0045111	intermediate filament cytoskeleton	2.85E-07	18
	GO:0005615	extracellular space	7.53E-07	62
	GO:0097060	synaptic membrane	1.51E-06	37
	GO:0005652	nuclear lamina	1.56E-06	8
	GO:0097381	photoreceptor disc membrane	9.17E-06	9
	GO:0043596	nuclear replication fork	9.57E-06	13
	GO:0043679	axon terminus	1.42E-05	28
	GO:0043195	terminal bouton	1.42E-05	23

**Ontology**	**ID**	**Description**	**qvalue**	**Count**
	**antagomir430c vs. antagomirNC**			
biological process	GO:0003299	muscle hypertrophy in response to stress	4.44E-14	18
	GO:0014887	cardiac muscle adaptation	4.44E-14	18
	GO:0014898	cardiac muscle hypertrophy in response to stress	4.44E-14	18
	GO:0003300	cardiac muscle hypertrophy	2.95E-12	25
	GO:0014897	striated muscle hypertrophy	3.33E-12	25
	GO:0014896	muscle hypertrophy	3.88E-12	25
	GO:0007215	glutamate receptor signaling pathway	1.22E-11	24
	GO:0014888	striated muscle adaptation	1.33E-11	20
	GO:0090201	negative regulation of release of cytochrome c from mitochondria	1.44E-11	15
	GO:0055012	ventricular cardiac muscle cell differentiation	2.13E-11	17
molecular function	GO:0071837	HMG box domain binding	5.10E-12	11
	GO:0008157	protein phosphatase 1 binding	1.41E-10	12
	GO:0003680	AT DNA binding	3.83E-09	9
	GO:0015605	organophosphate ester transmembrane transporter activity	1.28E-08	11
	GO:0004712	protein serine/threonine/tyrosine kinase activity	7.31E-08	11
	GO:0047555	3',5'-cyclic-GMP phosphodiesterase activity	5.38E-06	7
	GO:1990782	protein tyrosine kinase binding	5.53E-06	13
	GO:0008289	lipid binding	2.13E-05	35
	GO:0015026	coreceptor activity	5.13E-05	6
	GO:0047144	2-acylglycerol-3-phosphate O-acyltransferase activity	9.76E-05	6

cellular component	GO:0005882	intermediate filament	3.66E-12	17
	GO:0045111	intermediate filament cytoskeleton	4.02E-08	17
	GO:0005652	nuclear lamina	2.90E-07	8
	GO:0030016	myofibril	4.50E-07	26
	GO:0097381	photoreceptor disc membrane	9.75E-07	9
	GO:0099081	supramolecular polymer	1.37E-06	43
	GO:0099512	supramolecular fiber	1.37E-06	43
	GO:0043292	contractile fiber	1.98E-06	26
	GO:0044291	cell-cell contact zone	2.01E-06	13
	GO:0030017	sarcomere	8.60E-06	21
**Ontology**	**ID**	**Description**	**qvalue**	**Count**
	**antagomir430a vs. agomir430a**			
biological process	GO:0002931	response to ischemia	5.03E-18	22
	GO:0031554	regulation of DNA-templated transcription, termination	2.46E-14	11
	GO:0051941	regulation of amino acid uptake involved in synaptic transmission	2.46E-14	11
	GO:0051946	regulation of glutamate uptake involved in transmission of nerve impulse	2.46E-14	11
	GO:0060567	negative regulation of DNA-templated transcription, termination	2.46E-14	11
	GO:0032835	glomerulus development	2.05E-13	22
	GO:0097167	circadian regulation of translation	2.05E-13	11
	GO:0035983	response to trichostatin A	3.75E-13	10
	GO:0035984	cellular response to trichostatin A	3.75E-13	10
	GO:2001016	positive regulation of skeletal muscle cell differentiation	3.75E-13	10
molecular function	GO:1990226	histone methyltransferase binding	3.65E-12	11
	GO:0042826	histone deacetylase binding	3.65E-12	23
	GO:0001222	transcription corepressor binding	7.19E-11	11
	GO:0003680	AT DNA binding	2.43E-10	9
	GO:0071837	HMG box domain binding	7.56E-10	9
	GO:0015248	sterol transporter activity	7.35E-09	10
	GO:0036002	pre-mRNA binding	2.38E-08	11
	GO:0001221	transcription cofactor binding	1.20E-07	11
	GO:0004497	monooxygenase activity	2.27E-07	10
	GO:0005095	GTPase inhibitor activity	4.87E-07	7
cellular component	GO:0030485	smooth muscle contractile fiber	4.79E-10	10
	GO:0031092	platelet alpha granule membrane	6.97E-10	10
	GO:0097038	perinuclear endoplasmic reticulum	1.08E-09	9
	GO:0031091	platelet alpha granule	4.07E-05	11
	GO:0030667	secretory granule membrane	5.72E-05	18
	GO:0043292	contractile fiber	0.000161262	19
	GO:0099081	supramolecular polymer	0.000307435	30

Ontology	ID	Description	qvalue	Count
	GO:0099512	supramolecular fiber	0.000307435	30
	GO:0005790	smooth endoplasmic reticulum	0.001207213	6
	GO:0048471	perinuclear region of cytoplasm	0.001670913	32

**Ontology**	**ID**	**Description**	**qvalue**	**Count**
	**antagomir430b vs. agomir430b**			
biological process	GO:0055001	muscle cell development	6.86E-20	48
	GO:0055002	striated muscle cell development	6.86E-20	47
	GO:0030239	myofibril assembly	8.45E-16	30
	GO:0042692	muscle cell differentiation	1.67E-15	57
	GO:0061061	muscle structure development	7.50E-14	66
	GO:0098884	postsynaptic neurotransmitter receptor internalization	7.50E-14	12
	GO:0099590	neurotransmitter receptor internalization	7.50E-14	12
	GO:0140239	postsynaptic endocytosis	7.50E-14	12
	GO:1904978	regulation of endosome organization	7.50E-14	12
	GO:1904980	positive regulation of endosome organization	7.50E-14	12
molecular function	GO:1990175	EH domain binding	1.70E-13	12
	GO:0043812	phosphatidylinositol-4-phosphate phosphatase activity	1.05E-12	12
	GO:0016638	oxidoreductase activity, acting on the CH-NH2 group of donors	7.74E-11	11
	GO:0043813	phosphatidylinositol-3,5-bisphosphate 5-phosphatase activity	5.90E-10	12
	GO:0016641	oxidoreductase activity, acting on the CH-NH2 group of donors, oxygen as acceptor	6.30E-10	10
	GO:0071837	HMG box domain binding	6.30E-10	10
	GO:0052658	inositol-1,4,5-trisphosphate 5-phosphatase activity	2.07E-09	12
	GO:0003680	AT DNA binding	6.14E-09	9
	GO:0052629	phosphatidylinositol-3,5-bisphosphate 3-phosphatase activity	6.14E-09	12
	GO:0052659	inositol-1,3,4,5-tetrakisphosphate 5-phosphatase activity	6.14E-09	12
cellular component	GO:0030017	sarcomere	3.45E-20	41
	GO:0099081	supramolecular polymer	2.03E-19	69
	GO:0099512	supramolecular fiber	2.03E-19	69
	GO:0030016	myofibril	2.53E-18	42
	GO:0043292	contractile fiber	2.75E-18	44
	GO:0031674	I band	3.10E-12	25
	GO:0030132	clathrin coat of coated pit	2.14E-11	12
	GO:0030018	Z disc	2.56E-11	23
	GO:0005911	cell-cell junction	3.09E-09	49
	GO:0070161	anchoring junction	3.09E-09	49

Ontology	ID	Description	qvalue	Count
	**antagomir430c vs. agomir430c**			
biological process	GO:0030239	myofibril assembly	1.67E-14	46
	GO:0055002	striated muscle cell development	1.67E-14	71
	GO:0070848	response to growth factor	1.67E-14	135
	GO:0071363	cellular response to growth factor stimulus	1.67E-14	133
	GO:0055001	muscle cell development	1.67E-14	72
	GO:0030220	platelet formation	1.99E-14	23
	GO:0072010	glomerular epithelium development	7.17E-14	27
	GO:0036344	platelet morphogenesis	4.94E-13	24
	GO:0072160	nephron tubule epithelial cell differentiation	4.94E-13	24
	GO:0014706	striated muscle tissue development	2.23E-12	94
molecular function	GO:0042805	actinin binding	4.93E-12	28
	GO:0000976	transcription regulatory region sequence-specific DNA binding	6.09E-10	133
	GO:0000977	RNA polymerase II regulatory region sequence-specific DNA binding	1.98E-08	122
	GO:0004879	nuclear receptor activity	2.60E-07	26
	GO:0098531	transcription factor activity, direct ligand regulated sequence-specific DNA binding	2.60E-07	26
	GO:0042826	histone deacetylase binding	3.22E-07	37
	GO:0016316	phosphatidylinositol-3,4-bisphosphate 4-phosphatase activity	3.99E-07	10
	GO:0017161	inositol-1,3,4-trisphosphate 4-phosphatase activity	3.99E-07	10
	GO:0052828	inositol-3,4-bisphosphate 4-phosphatase activity	3.99E-07	10
	GO:0001216	bacterial-type RNA polymerase transcriptional activator activity, sequence-specific DNA binding	8.03E-07	84
cellular component	GO:0043292	contractile fiber	5.31E-19	77
	GO:0030017	sarcomere	5.34E-18	63
	GO:0030016	myofibril	9.22E-16	67
	GO:0031674	I band	4.72E-14	43
	GO:0030018	Z disc	4.72E-14	41
	GO:0099081	supramolecular polymer	4.48E-11	110
	GO:0099512	supramolecular fiber	4.48E-11	110
	GO:0071914	prominosome	4.25E-10	15
	GO:0070062	extracellular exosome	1.42E-08	31
	GO:1903561	extracellular vesicle	3.45E-08	31

Table S4 Significant KEGG enrichment among the DEGs.

Using *P* value to determine the significant value of the collected GO clusters, 0.05 represents significant significance. n=3 for each microinjection group.

**KEGG pathways significantly enriched by DEGs of agomir430a vs. agomirNC**			
**Pathway**	**map**	**DEGs**	**p value**
Steroid biosynthesis	malb00100	3	0.00044426257
Metabolic pathways	malb01100	19	0.00764973655
Starch and sucrose metabolism	malb00500	2	0.0365232931
Arachidonic acid metabolism	malb00590	2	0.0452261636
**KEGG pathways significantly enriched by DEGs of agomir430b vs. agomirNC**			
**Pathway**	**map**	**DEGs**	**p value**
MAPK signaling pathway	malb04010	17	0.0000392105
Protein processing in endoplasmic reticulum	malb04141	10	0.0004427799
C-type lectin receptor signaling pathway	malb04625	7	0.00201509974
Arachidonic acid metabolism	malb00590	4	0.005539994999
NOD-like receptor signaling pathway	malb04621	6	0.0204830636
Necroptosis	malb04217	6	0.023259437
Spliceosome	malb03040	5	0.0405685793
Histidine metabolism	malb00340	2	0.0426180703
Toll-like receptor signaling pathway	malb04620	4	0.0474010918
**KEGG pathways significantly enriched by DEGs of agomir430c vs. agomirNC**			
**Pathway**	**map**	**DEGs**	**p value**
Linoleic acid metabolism	malb00591	3	0.001816139
Thiamine metabolism	malb00730	2	0.01090177
Retinol metabolism	malb00830	3	0.015018061
Glutathione metabolism	malb00480	3	0.02243419
Pyrimidine metabolism	malb00240	3	0.029350102
Folate biosynthesis	malb00790	2	0.030235956
Tight junction	malb04530	6	0.042194682
beta-Alanine metabolism	malb00410	2	0.042721643
MAPK signaling pathway	malb04010	8	0.046248662
**KEGG pathways significantly enriched by DEGs of antagomir430a vs. antagomirNC**			
**Pathway**	**map**	**DEGs**	**p value**
Phototransduction	malb04744	9	9.89E-05
ECM-receptor interaction	malb04512	13	0.000742289
Tryptophan metabolism	malb00380	8	0.001078035
Apelin signaling pathway	malb04371	17	0.002950159
Cardiac muscle contraction	malb04260	13	0.003451457
Notch signaling pathway	malb04330	9	0.00451846
Metabolic pathways	malb01100	88	0.005191951
Adrenergic signaling in cardiomyocytes	malb04261	18	0.009234105
Nitrogen metabolism	malb00910	4	0.011138489
Alanine, aspartate and glutamate metabolism	malb00250	6	0.016480823

Porphyrin and chlorophyll metabolism	malb00860	5	0.016921894
Arginine biosynthesis	malb00220	4	0.022108979
Drug metabolism - other enzymes	malb00983	7	0.02644337
PPAR signaling pathway	malb03320	8	0.029958882
Calcium signaling pathway	malb04020	19	0.030181176
Ferroptosis	malb04216	6	0.035328767
Cytokine-cytokine receptor interaction	malb04060	15	0.03937936
Focal adhesion	malb04510	18	0.041670637
Fructose and mannose metabolism	malb00051	5	0.044083778

KEGG pathways significantly enriched by DEGs of antagomir430b vs. antagomirNC

Pathway	map	DEGs	p value
Cardiac muscle contraction	malb04260	27	1.22E-06
Drug metabolism - other enzymes	malb00983	16	5.74E-05
Adrenergic signaling in cardiomyocytes	malb04261	32	0.000269644
Steroid biosynthesis	malb00100	8	0.000304558
Steroid hormone biosynthesis	malb00140	12	0.000404661
Metabolic pathways	malb01100	142	0.001468612
Pyrimidine metabolism	malb00240	13	0.001503822
PPAR signaling pathway	malb03320	15	0.00152742
Tryptophan metabolism	malb00380	10	0.002280213
Arginine biosynthesis	malb00220	7	0.002591061
Alanine, aspartate and glutamate metabolism	malb00250	10	0.002612997
Nitrogen metabolism	malb00910	6	0.003770627
Phototransduction	malb04744	8	0.009315894
Retinol metabolism	malb00830	9	0.013195618
Neuroactive ligand-receptor interaction	malb04080	49	0.014673913
ECM-receptor interaction	malb04512	14	0.018014478
Metabolism of xenobiotics by cytochrome P450	malb00980	7	0.020908087
Adipocytokine signaling pathway	malb04920	13	0.022966178
Pantothenate and CoA biosynthesis	malb00770	4	0.023129897
Drug metabolism - cytochrome P450	malb00982	7	0.023369916
Purine metabolism	malb00230	19	0.029739991
beta-Alanine metabolism	malb00410	6	0.033037439
Biosynthesis of amino acids	malb01230	11	0.035171034
Calcium signaling pathway	malb04020	28	0.038279632
ABC transporters	malb02010	7	0.038677602

KEGG pathways significantly enriched by DEGs of antagomir430c vs. antagomirNC

Pathway	map	DEGs	p value
Cardiac muscle contraction	malb04260	23	3.70E-07
ECM-receptor interaction	malb04512	16	0.000136255
Adrenergic signaling in cardiomyocytes	malb04261	25	0.00031513
Notch signaling pathway	malb04330	12	0.000460756
Apelin signaling pathway	malb04371	21	0.000642066
Tight junction	malb04530	25	0.001633437

Focal adhesion	malb04510	26	0.001645582
Glycerolipid metabolism	malb00561	11	0.001968871
Pyrimidine metabolism	malb00240	10	0.002800079
Phototransduction	malb04744	7	0.005112265
Calcium signaling pathway	malb04020	24	0.008335046
Cell adhesion molecules (CAMs)	malb04514	18	0.008940984
Porphyrin and chlorophyll metabolism	malb00860	6	0.008997557
Metabolic pathways	malb01100	99	0.011734746
Apoptosis	malb04210	16	0.022895353
Biosynthesis of unsaturated fatty acids	malb01040	5	0.041087722
Nicotinate and nicotinamide metabolism	malb00760	5	0.041087722

KEGG pathways significantly enriched by DEGs of antagomir430a vs. agomir430a

Pathway	map	DEGs	p value
Ubiquinone and other terpenoid-quinone biosynthesis	malb00130	4	0.001639767
ECM-receptor interaction	malb04512	11	0.001905114
Biosynthesis of unsaturated fatty acids	malb01040	6	0.002916499
Metabolism of xenobiotics by cytochrome P450	malb00980	6	0.003283144
Steroid hormone biosynthesis	malb00140	6	0.010785374
Tyrosine metabolism	malb00350	5	0.019910623
Metabolic pathways	malb01100	72	0.024975814
Phenylalanine metabolism	malb00360	3	0.027814547
Vitamin B6 metabolism	malb00750	2	0.034418306
Nitrogen metabolism	malb00910	3	0.035775339
Fatty acid elongation	malb00062	4	0.043877112
Steroid biosynthesis	malb00100	3	0.044776685
Base excision repair	malb03410	4	0.047383004

KEGG pathways significantly enriched by DEGs of antagomir430b vs. agomir430b

Pathway	map	DEGs	p value
Focal adhesion	malb04510	44	1.09E-10
ECM-receptor interaction	malb04512	23	2.22E-08
MAPK signaling pathway	malb04010	46	6.88E-07
Glycolysis / Gluconeogenesis	malb00010	13	0.000549043
Regulation of actin cytoskeleton	malb04810	28	0.000583536
AGE-RAGE signaling pathway in diabetic complications	malb04933	18	0.000872982
Cardiac muscle contraction	malb04260	15	0.002485621
C-type lectin receptor signaling pathway	malb04625	15	0.00324299
Adrenergic signaling in cardiomyocytes	malb04261	22	0.003455064
Tight junction	malb04530	24	0.003652087
Carbon metabolism	malb01200	15	0.004725827
Pentose phosphate pathway	malb00030	7	0.005378247
Insulin signaling pathway	malb04910	18	0.00548498
Apoptosis	malb04210	18	0.006377911
ErbB signaling pathway	malb04012	13	0.006542007

Histidine metabolism	malb00340	5	0.009654529
Salmonella infection	malb05132	10	0.014707035
Glycerolipid metabolism	malb00561	9	0.016098175
Wnt signaling pathway	malb04310	18	0.01711892
Biosynthesis of amino acids	malb01230	9	0.027275596
Calcium signaling pathway	malb04020	22	0.028105492
RNA degradation	malb03018	9	0.032611284
Glycine, serine and threonine metabolism	malb00260	6	0.03693205
Necroptosis	malb04217	14	0.040370174
Phagosome	malb04145	13	0.046859549
Thiamine metabolism	malb00730	3	0.047284507
Adherens junction	malb04520	11	0.049396371

KEGG pathways significantly enriched by DEGs of antagomir430c vs. agomir430c

**Pathway**	**map**	**DEGs**	**p value**
Focal adhesion	malb04510	80	2.53E-09
ECM-receptor interaction	malb04512	38	1.10E-07
Ribosome	malb03010	38	7.06E-05
MAPK signaling pathway	malb04010	90	0.000110822
Cellular senescence	malb04218	52	0.000591355
Calcium signaling pathway	malb04020	73	0.001982638
Cytokine-cytokine receptor interaction	malb04060	50	0.002299178
Cysteine and methionine metabolism	malb00270	17	0.003089777
Regulation of actin cytoskeleton	malb04810	60	0.004003949
Thiamine metabolism	malb00730	7	0.00439706
TGF-beta signaling pathway	malb04350	32	0.004436177
Cardiac muscle contraction	malb04260	31	0.005764295
Tight junction	malb04530	54	0.008417078
Adrenergic signaling in cardiomyocytes	malb04261	48	0.009145155
Tyrosine metabolism	malb00350	12	0.015460561
Apelin signaling pathway	malb04371	39	0.017057461
ABC transporters	malb02010	12	0.022776743
FoxO signaling pathway	malb04068	40	0.026808131
2-Oxocarboxylic acid metabolism	malb01210	7	0.034110126
Biosynthesis of amino acids	malb01230	19	0.0394327
Cell adhesion molecules	malb04514	39	0.040818826
Melanogenesis	malb04916	29	0.055688496
Phenylalanine metabolism	malb00360	5	0.089395287
Adherens junction	malb04520	24	0.096363946
Vascular smooth muscle contraction	malb04270	33	0.099185464
Endocytosis	malb04144	59	0.112018869
Glycosphingolipid biosynthesis - lacto and neolacto series	malb00601	8	0.112175222
p53 signaling pathway	malb04115	19	0.114533993
Tryptophan metabolism	malb00380	10	0.120082573

Necroptosis	malb04217	31	0.123448035
ErbB signaling pathway	malb04012	22	0.123527747
Adipocytokine signaling pathway	malb04920	19	0.124062783
Starch and sucrose metabolism	malb00500	9	0.124687236
Citrate cycle (TCA cycle)	malb00020	8	0.14701619
Alanine	malb00250	10	45/6578
Nitrogen metabolism	malb00910	5	0.161303821
Gap junction	malb04540	22	0.172877332
VEGF signaling pathway	malb04370	15	0.179754464
Wnt signaling pathway	malb04310	39	0.189744606
Hedgehog signaling pathway	malb04340	13	0.201632559
Insulin signaling pathway	malb04910	32	0.20929526
Mucin type O-glycan biosynthesis	malb00512	8	0.22983908
Glyoxylate and dicarboxylate metabolism	malb00630	8	0.22983908
Lysine degradation	malb00310	14	0.236945311
GnRH signaling pathway	malb04912	22	0.256668033
Intestinal immune network for IgA production	malb04672	6	0.262148525
Arginine biosynthesis	malb00220	5	0.281322886
Ubiquinone and other terpenoid-quinone biosynthesis	malb00130	3	0.283220439
Other types of O-glycan biosynthesis	malb00514	10	0.283255315

DEG, differentially expressed gene.

Table S5 Primers used for real-time PCR analysis.

Gene	GenBank accession No.	Forward primer (5’-3’)	Reverse primer (5’-3’)
18s rRNA	LC151290	GCTCGTAGTTGGATCTCGGG	ACCTCTAGCGGCACAATACG
ef1a	KC011266	ATTGATGCCCCTGGACACAG	TGTAGGCGCTCACTTCCTTG
gapdh	XM_020610927	CTTCCTGCACAACCAACTGC	TTGCCGTTGAGCTCAGGAAT
β-actin	AY345056	TCAACACGCCTGCCATGTAT	CGCTCAGCTGTGGTAGTGAA
hprt	DQ218476	TTTGTGGTGGGATATGCGCT	CTGTCAGGATTGTCGCCACT
rpl17	XM_020587712	TCCCTTTCCGTCGCTACAAC	CTCATCTTGGGGGCCTTGTT
3β-hsd	XM_020586257	GATGGGACCAAACTCCAGGG	ACGCATATGGCCCAAAAGGA
amh	KF770790	TCAGCAAAAGCAGCTTCCAC	TTCCAAACAGCGCCAAACTG
ar	FJ471538	CCGTATGAGAGGAGCGTCAC	TCTGCAGCTCGCTTGAAGAA
cyp11a1	XM_020619382	CTGAAGCGGATTCCCTTGGT	GGTCGATACTGCTCTGGACG
cyp17	AY224681	GGTCCCCCAAGCTTAGTGAC	AGATAGCTGGGTGATGGGGT
cyp19a1a	EU252487	AAAATGCTCCTCGCCGTTAC	TCACCATGGCAATGTGCTTG
cyp19a1b	EU252488	TCAATGCGAAGGACTTGCTG	TGTTGCACAGCCATCCAATC
dax1	KF770791	CTGTTTTCGTGAGTTGCCGG	TGGCTTCGATTTCCACGACA
dmrt1	AF421347	TCTCAGTACCGCATGCATTC	TGTTGTTGTTGCTGCTGCTG
era	KM039134	AAATCGGAGGAAGAGCTGCC	CTCCAGGTCCTTAGAGGCCT
erb	FJ873737	CCCTCACAGAGCAGAACTGG	GGAATCTTCTTGGCCCAGCT

foxl2	KC470042	GAGAAGCGCCTCACTCTCTC	AACAAGGACTTACCCGGCTG
foxl3		GGGACAGGAAGGGCAACTAC	AGTGATGACCTGGTGTGCTG
hsd11b2	XM_020602679	CGGATCGTCACCATCTCCAG	TTGAACGCAGATGGCAGGAT
hsd17b1	XM_020607406	GAACGTTAGTGAGCTGGGCT	CAACCCTGTCCCTCACATCC
hsd17b2	XM_020615849	TGACTGACGGCTCCCAGATA	GCTCACGTTGATGATGCGTC
hsd17b3	XM_020593846	ATGCCACAGGACAGAAGGTG	ATTGTCTGCTCCAGGTCTGC
hsd17b4	XM_020593869	ACAGATGCCACCAAGCCTAC	ACCCCCAGTGCATAGAGGAT
hsd17b7	XM_020585820	CACCTTGATGTGGGCTCTGT	AACTTCCTGCAGACCGTCTG
hsd17b8	XM_020587627	CGATACTTCCAGCCTCCGTC	GGTTAAACCCTCCACTCCGG
hsd17b10	XM_020586601	ATGTGATTCGCCTTGCTGTG	ATCAAATGCTGCCACACTGG
hsd17b12	XM_020623188	ACATGACCTCAGTGTGCCAG	GAAAGGCAGCACGCTTTGAA
hsd17b14	XM_020605399	TTCCATGACAAAGGCGATGG	TTAATGGCAGCTGCAGCATC
jnk	EF661977	AAGGTGATCGAGCAGTTGGG	GCCTCGTCCACTGAGATACG
sf-1	KJ131498	AGCCTCTCATGCTGGTCCTA	CAGACAGACACAGCAGCAGA
sox3	XM_020592532	AAGTATTCTTTGCCCGGGGG	CATCATCGGGTACTGGAGCC
sox9	XM_020622629	CGTCAGACAACCACCTCCTC	TTTTGCTGAGTTCCGCGTTG
star	XM_020590513	ATGGAGCAAATGGGGGAGTG	CTACACCCTTCTGCTCAGGC
vasa	AY912131	GCCAGGAAGTTTGCCTTTGG	TGATTCTCTTTGGGCGGCAT
wt1	KC015038	GACACTGACCCTCACTGTGG	ACCGTGCTGTAACCTGTCTG
miR430a-stem-loop		GTCGTATCCAGTGCAGGGTCCGAGGTATTCGCACTGGATACGACctaccc	
miR430b-stem-loop		GTCGTATCCAGTGCAGGGTCCGAGGTATTCGCACTGGATACGACctaccc	
miR430c-stem-loop		GTCGTATCCAGTGCAGGGTCCGAGGTATTCGCACTGGATACGACcaaccc	
miR-430a		CGCGCTAAGTGCTATTTGTTG	GTGCAGGGTCCGAGGT
miR-430b		GCGCAAAGTGCTATCAAGTTG	GTGCAGGGTCCGAGGT
miR-430c		CGCGCTAAGTGCTTCTCTTTG	GTGCAGGGTCCGAGGT

Table S6 RNA sample purity and integrity.

"RIN" = RNA Integrity Number, A260/A280/A230 = RNA sample absorbances at wavelengths 260 nm, 280 nm, and 230 nm, respectively.

Sample nuber	RNA purity		RNA integrity	
	A260/280	A260/230	RIN	28S/18S
agomirNC-1	2.1	1.7	10.0	1.8
agomirNC -2	2.1	1.6	10.0	1.8
agomirNC -3	2.1	1.6	10.0	1.8
agomir430a-1	2.1	1.6	10.0	1.7
agomir430a -2	2.1	1.9	10.0	1.7
agomir430a -3	2.0	1.6	10.0	1.7
agomir430b-1	1.9	2.2	10.0	1.8
agomir430b-2	2.0	1.7	10.0	2.0

agomir430b-3	2.1	1.6	10.0	2.0
agomir430c-1	2.0	1.7	10.0	1.9
agomir430c-2	2.1	1.9	10.0	1.9
agomir430c-3	2.0	1.7	10.0	2.0
antagomirNC-1	2.1	1.2	9.6	1.4
antagomirNC-2	2.1	1.4	10.0	1.6
antagomirNC-3	2.1	0.7	10.0	1.6
antagomir430a-1	2.2	0.6	9.1	1.4
antagomir430a-2	2.2	0.8	10.0	1.6
antagomir430a-3	2.2	1.0	10.0	1.6
antagomir430b-1	2.1	1.4	9.9	1.5
antagomir430b-2	2.1	1.2	10.0	1.6
antagomir430b-3	2.1	0.9	10.0	1.5
antagomir430c-1	2.1	0.7	10.0	1.5
antagomir430c-2	2.1	1.6	10.0	1.5
antagomir430c-3	2.2	1.3	10.0	1.6

Table S7 steroidogenesis-related gene expression in RNA-Seq.

Gene	FC/log2FC					
	T11 vs C1	T12 vs C1	T13 vs C1	T21 vs C2	T22 vs C2	T23 vs C2
star	1.01/0.01	0.80/-0.32	0.83/-0.27	0.36/-1.46*	0.27/-1.88**	0.66/-0.61
cyp17a1	0.84/-0.26	0.50/-0.99	1.04/0.06	0.42/-1.27	0.74/-0.422	0.48/-1.07
sf-1	0.61/-0.7	1.06/0.08	0.76/-0.40	0.69/-0.54	0.85/-0.24	0.69/-0.53
wt1	0.93/-0.10	0.90/-0.15	1.10/0.14	0.82/-0.28	1.04/0.06	0.92/-0.12
cyp19a1a	0.23/-2.15	0.61/-0.71	1.66/0.73	0.53/-0.92	0.19/-2.42	0.24/-2.07
cyp19a1b	1.34/0.42	1.67/0.74*	1.36/0.44	0.51/-0.96*	0.46/-1.11	0.40/-1.32**
sox3	1.10/0.14	1.03/0.05	0.84/-0.26	1.34/0.42	1.21/0.27	1.17/0.23
jnk1	0.98/-0.03	1.00/0.01	1.00/0.00	0.89/-0.16	1.16/0.21	1.10/0.13
foxl2	0.88/-0.19	1.04/0.06	0.98/-0.03	1.40/0.48	1.28/0.35	1.44/0.52
dmrt1	5.73/2.52	5.41/2.44	7.29/2.86	0.14/-2.79	0.14/-2.85	0.51/-0.96
amh	1.13/0.18	1.01/0.02	0.88/-0.19	1.62/0.70	2.03/1.02	1.33/0.41

sox9	0.92/-0.12	0.99/-0.02	0.92/-0.12	1.39/0.47	1.30/0.37	1.30/0.38
foxl3	0.97/-0.04	0.98/-0.03	1.07/0.09	0.99/-0.01	1.48/0.57	1.06/0.08
dax1	1.21/0.28	1.61/0.68	1.57/0.65	0.67/-0.58	0.43/-1.21**	0.56/-0.84*
hsd17b14	1.01/0.02	1.13/0.17	1.25/0.33	1.27/0.35	1.13/0.18	1.01/0.01
hsd17b1	5.21/2.38	1.30/0.38	0.67/-0.58	0.41/-1.30	0.97/-0.04	0.61/-0.72
HSD17B7	1.28/0.35	1.45/0.53	1.11/0.15	2.13/1.09*	2.25/1.17**	1.98/0.98*
3Beta_HSD	0.63/-0.67	0.79/-0.34	0.80/-0.32	0.56/-0.83*	0.72/-0.47	0.53/-0.91*
hsd17b12	1.08/0.12	1.11/0.15	0.97/-0.20	0.46/-1.11**	0.65/-0.62	0.35/-1.52**
hsd17b10	0.82/-0.28	0.98/-0.03	0.97/-0.04	0.95/-0.08	1.19/0.25	0.92/-0.12
hsd17b8	1.29/0.37	0.76/-0.40	0.95/-0.07	1.39/0.47	1.01/0.01	1.41/0.50
hsd17b2	1.53/0.61*	1.21/0.27	0.97/-0.05	0.56/-0.84*	0.60/-0.75*	1.01/0.02
hsd17b3	1.66/0.73	0.52/-0.93	0.55/-0.87	0.19/-2.43	0.08/-3.67**	0.20/-2.35**
hsd17b4	0.90/-0.15	0.99/-0.01	1.12/0.08	1.02/0.03	0.95/-0.08	0.96/-0.05
hsd11b2	0.97/-0.05	0.97/-0.05	1.05/0.08	0.82/-0.29	0.77/-0.39	0.78/-0.35
cyp11a1	1.05/0.07	0.73/-0.46	1.01/0.01	0.68/-0.56	0.80/-0.32	1.48/0.56
era	0.96/-0.06	0.82/-0.29	1.05/0.07	0.86/-0.21	1.50/0.58	1.17/0.22
erb	1.20/0.27	1.01/0.02	1.19/0.25	0.79/-0.34	0.98/-0.03	0.70/-0.52
ar	0.84/-0.26	1.08/0.11	1.07/0.10	0.78/-0.35	0.56/-0.84	0.65/-0.63
vasa	0.86/-0.22	0.88/-0.19	0.96/-0.06	0.33/-1.59*	0.59/-0.77	0.42/-1.25*

Note: FC, FoldChange; log2FC, log2Foldchange. * represented FC>1.5 and P<0.05; ** represented FC>1.5, P<0.05 and FDR-adjusted P<0.05.

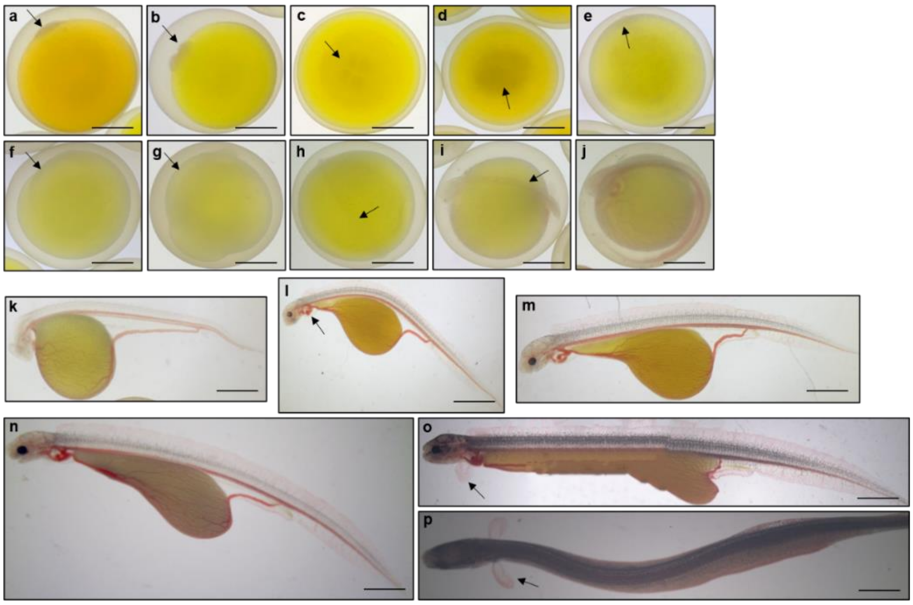

Figure S1. External appearance of *M. albus* embryos in different periods. (a) zygote: blastodisc formation; (b, c) cleavage: 2-cell, 4-cell; (d) blastula stage; (e, f, g, h, i) gastrula: shield, gastrula telophase, neurula metaphase, blastopore closing, tail bud formation; (j, k) hatching prophase: 4 d after fertilization; (l, m, n, o, p) hatching: hatching (newly hatching fry), 1 d after hatching, 2 d after hatching, 4 d after hatching, 6 d after hatching. The arrows represented (a-e) blastodermal cells, (f-h) gastrula, (i) appearance of tail bud, (l) heart, (o, p) pectoral fin. Scale bar = 1.5 mm.

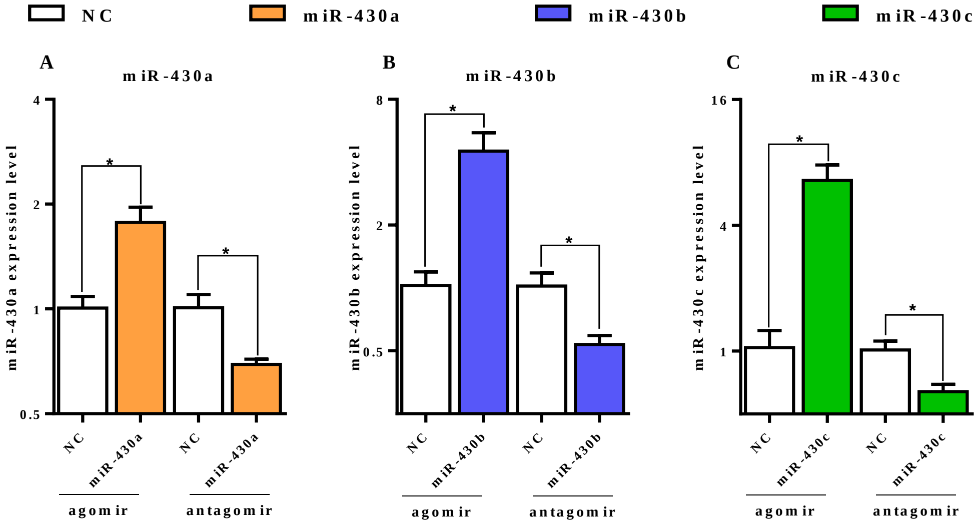

Figure S2. The miR-430 expression levels of *M. albus* after microinjection. (A) miR-430a; (B) miR-430b; (C) miR-430c. The results of qRT-PCR were performed by relative expression using U6 snRNA as the reference gene and measured by the method of optimized comparative Ct ($2^{-\Delta\Delta Ct}$) value. NC stands for negative control. Values are means ± SEM., n=3. * $P \leqslant 0.05$ (Student's t-test).

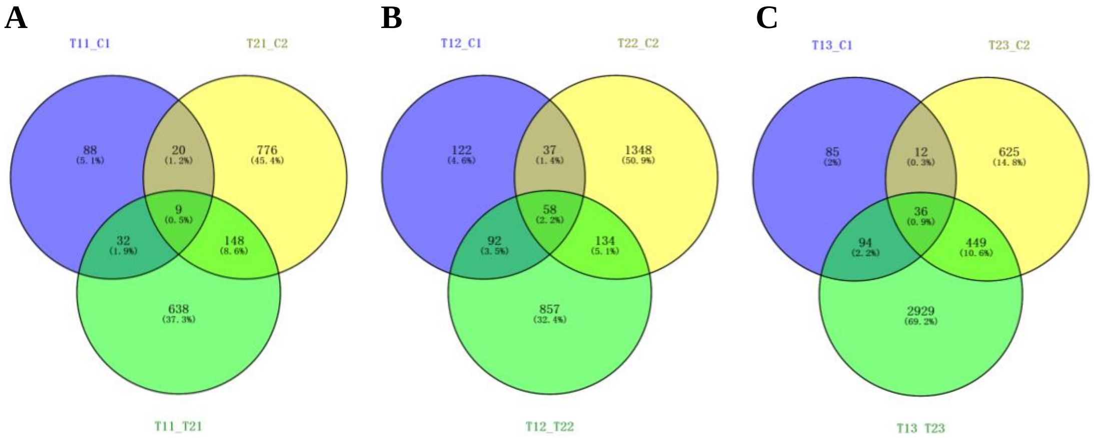

Figure S3. Venn diagram showing the number of significantly differential expressed genes identified by pairwise comparisons of different groups. (A) miR-430a; (B) miR-430b; (C) miR-430c. C1, agomirNC; C2, antagomirNC; T11, agomir430a; T21, antagomir430a; T12, agomir430b; T22, antagomir430b; T13, agomir430c; T23, antagomir430c. n=3 for each microinjection group.

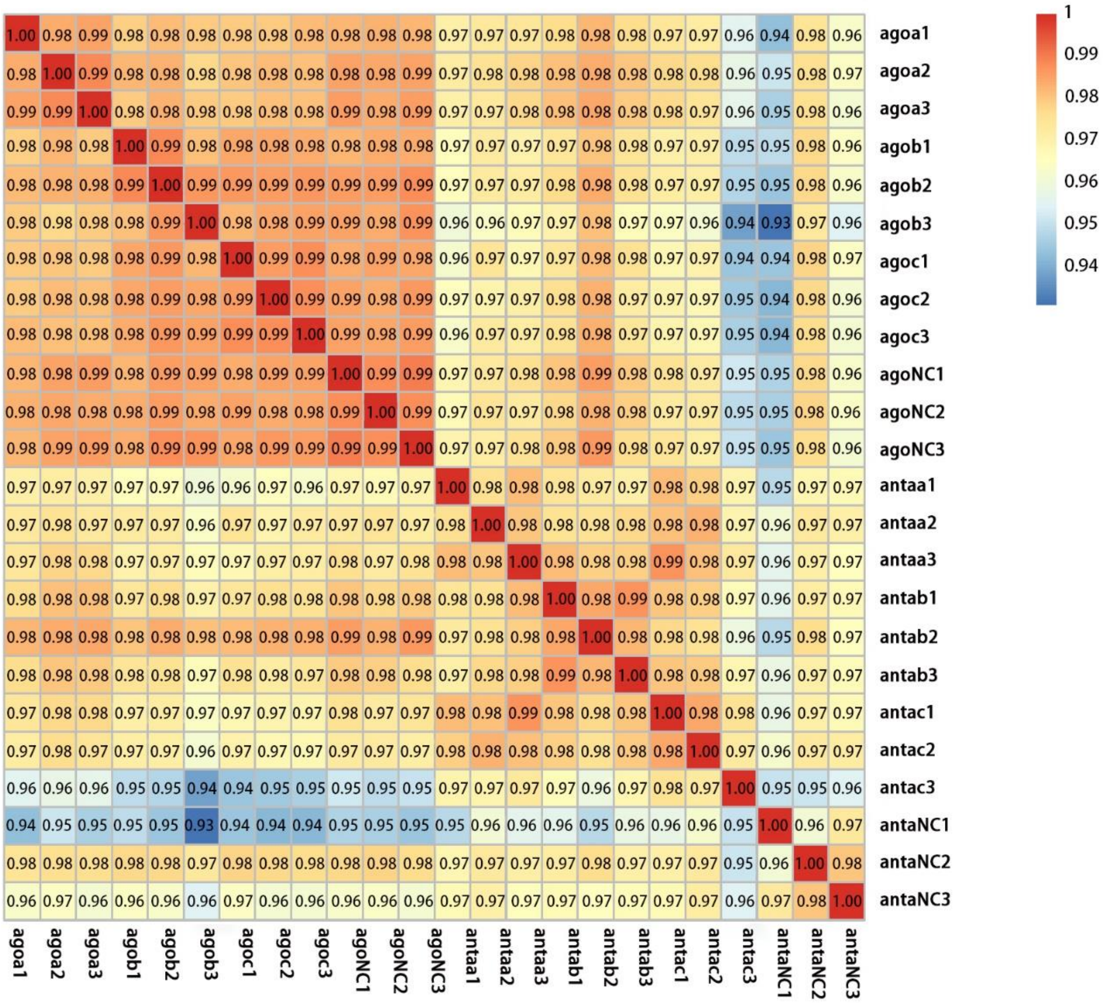

Figure S4. Heatmap of the coefficient matrix for the species-abundance clustering image. Values near 1 represent the smallest distances between the three sequence samples for each treatment.

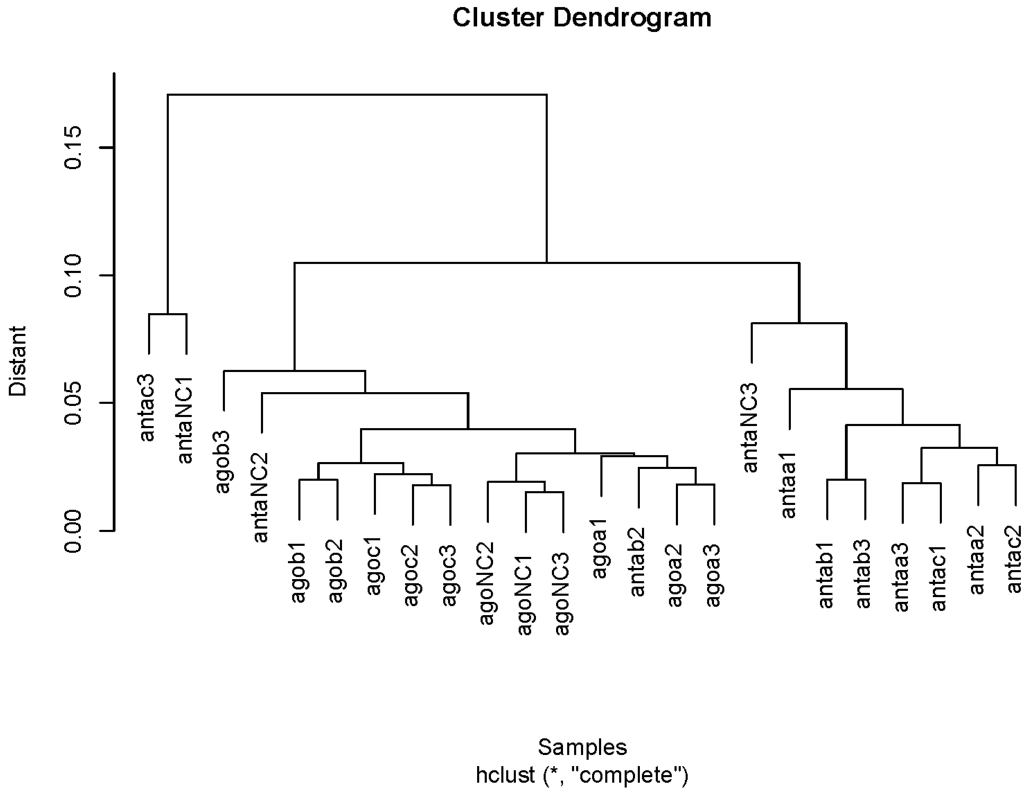

Figure S5. Cluster dendrogram of the sample-to-sample distances for the species-abundance clustering image.

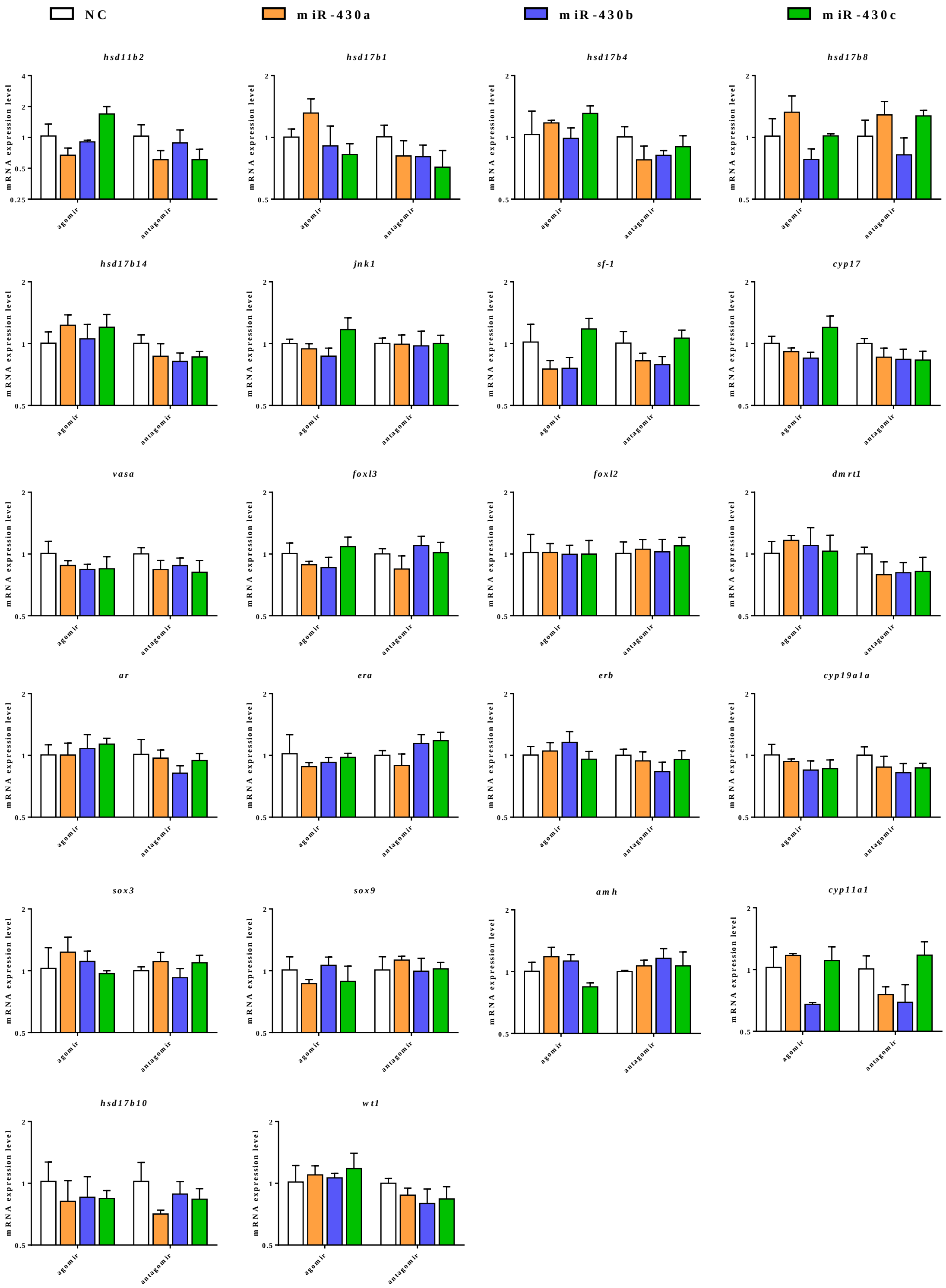

Figure S6. Candidate genes expression levels revealed by quantitative qPCR. The results of qPCR were performed by relative expression using *β-actin* and *ef1a* as the reference gene and measured by the method of optimized comparative Ct (2-ΔΔCt) value. Values are means ± SEM., n=3. *$p < 0.05$.
